# Glucose Regulates Glucose Transport and Metabolism via mTOR Signaling Pathway in Bovine Placental Trophoblast Cells

**DOI:** 10.3390/ani14010040

**Published:** 2023-12-21

**Authors:** Liyuan Shi, Kun Kang, Zhisheng Wang, Junmei Wang, Jianxin Xiao, Quanhui Peng, Rui Hu, Jia Zhou, Xiaohong Zhang, Ziqi Yue, Huawei Zou, Bai Xue, Lizhi Wang

**Affiliations:** Low Carbon Breeding Cattle and Safety Production University Key Laboratory of Sichuan Province, Animal Nutrition Institute, Sichuan Agricultural University, Chengdu 611130, China; sly2021214030@126.com (L.S.); kangk2017@163.com (K.K.); junmeiwangsicau@163.com (J.W.); xiaojianxin@sicau.edu.cn (J.X.); pengquanhui@126.com (Q.P.); ruitianhu@yeah.net (R.H.); 2019114010@stu.sicau.edu.cn (J.Z.); zhangxiaohongsicau@163.com (X.Z.); yzq947036981@163.com (Z.Y.); zhwbabarla@126.com (H.Z.); xuebai@sicau.edu.cn (B.X.); 12825@sicau.edu.cn (L.W.)

**Keywords:** BPTCs, glucose, glucose transport, glucose metabolism, mTOR

## Abstract

**Simple Summary:**

During pregnancy, especially the perinatal period, cows are often in a state of negative energy balance due to the imbalance of glucose metabolism. As the main energy substrate to promote fetal development, glucose is mainly obtained from the mother through the placenta and the provision of glucose to the fetus depends on the activity of glucose transporters distributed within the placental tissue. As the basic component of placental tissue, placental trophoblast cells participate in nutrient supply and metabolism between mother and fetus. However, a noticeable void exists in the study of the function and potential regulation mechanism of placental trophoblast cells in ruminants. In light of this, the bovine placental trophoblast cells were treated with different dose-concentration glucose and the gene expression of glucose transport carriers and enzymes related to glucose metabolism were detected. Moreover, the signaling pathway of placental-sensing energy level changes were studied to explore the mechanism of placenta mediating energy metabolism transmission between mother and fetus. The results showed that glucose regulates cellular glucose transport and metabolism by mediating the mTOR signaling pathway in bovine placental trophoblast cells.

**Abstract:**

It has been confirmed that improving the energy level of the diet contributed to the greater reproductive performance and birth weight of calves in periparturient dairy cows. To investigate the effect of glucose on nutrient transport during fetal development, the bovine placental trophoblast cells (BPTCs) were cultured in media with different glucose concentrations (1, 2, 4, 8, or 16 mg/mL). Subsequently, the BPTCs were cultured in media with 1, 8 mg/mL glucose and 8 mg/mL glucose plus 100 nmol/L rapamycin (the inhibitor of mTOR pathway). Compared with the 1 mg/mL glucose, the addition of 8 mg/mL glucose stimulated cell proliferation, upregulated the mRNA abundance of the glucose transporter GLUT1 and GLUT4, and increased the activity of glucose metabolism-related enzyme glucose-6-phosphate dehydrogenease (G6PD), lactate dehydrogenase (LDHA) and phosphoglycerate kinase 1 (PGK1), as well as adenosine-triphosphate (ATP) content (*p* < 0.05).Furthermore, compared with the treatment of 1 mg/mL glucose, adding 8 mg/mL of glucose-upregulated gene expression in the mTOR signaling pathway, including phosphatidylinositol3-kinase (PI3K), protein kinase B (Akt), mammalian target of rapamycin (mTOR) and 70 kDa ribosomal protein S6 kinase 2 (P70S6K) (*p* < 0.05).The supplementation of rapamycin downregulated the gene and protein expression of the mTOR signaling pathway, including mTOR, P70S6K, EIF4E-binding protein 1 (4EBP1), hypoxia-inducible factor 1-alpha (HIF-1α) and gene expression of glucose transporter upregulated by 8 mg/mL glucose (*p* < 0.05). Thus, these results indicated that the addition of 8 mg/mL glucose regulated the glucose transport and metabolism in BPTCs through the mTOR signaling pathway, thereby promoting the supply of nutrients to fetus.

## 1. Introduction

Glucose is the main source of energy for mammalian cells, serving as an indispensable nutrient for sustaining animal life and ensuring productivity. Like other essential nutrients, glucose has the ability to directly regulate gene expression within cells [1]. During the gestation of cows, particularly in the transition period, there are more energy requirements than in other physiological states. The energy from the diet is not only used for its own maintenance needs but is also supplied to the fetus through the blood and placenta. Consequently, most pregnant cows usually experience a state of negative energy balance during this critical period. In recent years, research has suggested that reduced glycemia may be among the causes of metabolic diseases during the transition period which lead to health, production, and reproductive problems in cows [2,3]. Therefore, increasing the supply of glucose during pregnancy is essential to meet the energy demands of cows and fetuses.

The placenta is an important hub between the mother and the fetus, ensuring an adequate supply of nutrients to the fetus during pregnancy [4]. The placental trophoblast cells (PTC) are located in the placental tissue and are responsible for the exchange of nutrients between the mother and fetus. The application of PTC focused on the study of extensive fields such as pregnancy recognition, embryo implantation, placental formation, fetal development, and pregnancy maintenance [5,6,7]. The glucose transporters (GLUTs) are the main transporters of glucose uptake by the PTC [8,9], and the glucose transporter 1 (GLUT1) [10], glucose transporter 3 (GLUT3) [11,12], and glucose transporter 4 (GLUT4) are highly expressed in placental tissue [13]. Our recent in vivo study showed that the mRNA abundance of GLUT1 and GLUT3 in placental tissue increased with the increase of dietary energy levels during pregnancy and was positively correlated with the birth weight and serum glucose concentration in newborn calves [14]. The primary glucose transporters in the placenta consist of GLUT1 and GLUT3, both of which regulate the transportation of glucose from the maternal to the fetal circulation [15,16]. There are also a series of nutrient transport sensing signal pathways in the placenta, such as mammalian target of rapamycin (mTOR). The mTOR signal pathway regulates cell growth and metabolic state through sensing nutrients, growth factors, and extracellular signals [17,18,19] with mTOR Complex 1 (mTORC1) and mTOR Complex 2 (mTORC2) [20]. A previous study demonstrated that the exogenous addition of glucose to human primary placental trophoblast cells can alter the gene expression of amino acid transporters through the mTOR pathway [21].

The close relationship between the glucose concentration in maternal blood and fetal growth and offspring development has been extensively demonstrated in humans and mice [22,23]. However, due to the unique economic characteristics of cows, research on pregnant cows has focused on the balanced supply of glucose and its impact on milk production [24]. To our knowledge, nutrition and the regulation of maternal glucose are crucial for the optimal growth and development of offspring, but the mechanism of glucose on nutrient transport and glucose metabolism in bovine placental trophoblast cells is still unclear. Therefore, in this study, the bovine placental trophoblast cells (BPTCs) model was used to explore the effect of glucose on nutrient transport and glucose metabolism in the placenta.

## 2. Materials and Methods

### 2.1. Cell Culture

The BPTCs were provided by the Beijing Academy of Agricultural Sciences (Beijing, China) [25] and cultured in a DMEM/F-12 medium (Gibco, Grand Island, NE, USA) supplemented with 10% fetal bovine serum (Gibco, Grand Island, NE, USA) and 1% triple antibiotics (100 IU/mL penicillin, 10 µg/mL streptomycin, 25 µg/mL amphotericin B; SolelyBio, Beijing, China). The culture conditions were 37 °C and 5% CO_2_, which is according to the pre-established culture conditions in the laboratory.

### 2.2. BPTCs Growth Curve

Approximately 2000 cells in 100 μL medium were added to a 96-well plate (Wuxi NEST Biotechnology Co., Ltd., Wuxi, China). Following a 24 h period of adhesion, 10 μL of CCK8 (Cell Counting Kit-8, Abmole, Shanghai, China) was added to the designated wells for daily detection. To ensure accuracy, six parallel wells were set up per counting point. The microplate was incubated at 37 °C and 5% CO_2_ for 1 h and then the optical density value at 450 nm (OD450) was detected by a microplate reader (Bio-Rad, Hercules, CA, USA). This process was repeated for a total of 7 days. The measurement results were plotted on a graph with the culture time on the abscissa and OD450 on the ordinate, generating a growth curve for BPTCs.

### 2.3. Immunofluorescence Identification of BPTCs

Approximately 4 × 10^5^ cells in 2 mL medium were inoculated in a 6-well plate (NEST) to 80% confluency observe cell morphology by an inverted microscope. Then, 1 mL of 4% paraformaldehyde (*v*/*v*, Beyotime Biotechnology, Shanghai, China) was added to each well at room temperature to fix the cells for 30 min. Then, the cells were blocked with 3% goat serum (Beyotime Biotechnology, Shanghai, China) for 60 min and incubated overnight at 4 °C with the anti-cytokeratin 7 (CK7, 1:100, Proteintech, Wuhan, China). Subsequently, the cells were incubated with the secondary antibody of goat anti-mouse IgG combined with fluorescein isothiocyanate (FITC) at 37 °C for 60 min. The 4’,6-diamino-2-phenylindole (DAPI, Solarbio, Beijing, China) were used to label the nucleus and then observed with a fluorescence microscopy (Nikon, Japan). Phosphate-buffered saline (PBS, Gibco, Grand Island, NE, USA) was used to wash the cells three times for 5 min after each step.

### 2.4. Experimental Design

The BPTCs at 80% confluency was transferred into 6-well plates or 96-well plates (Wuxi NEST Biotechnology Co., Ltd., Wuxi, China). When the cell confluency reached 80%, the medium (Pronox, Wuhan, China) was changed to containing 1, 2, 4, 8, and 16 mg/mL of glucose (D-glucose, ≥99.5%, Sigma, St. Louis, MO, USA) for 24 h. The BPTCs were inoculated and reached 80% confluency and starved with glucose-free DMEM/F-12 medium for 6 h. Another set of treatment is carried out based on the appropriate concentration screened. The BPTCs incubated in the glucose-free DMEM/F-12 medium contain 1 or 8 mg/mL glucose with or without the addition of 100 nmol/L rapamycin for 24 h to evaluate the effects of the glucose transporter, metabolism, and the proteins expression in mTOR signaling pathway.

### 2.5. Cell Morphology and Viability Assay

The BPTCs were inoculated in 96-well plates; after this, the cells would grow adductively and then treated with 200 µL medium (Pronox, Wuhan, China) containing different concentrations 1, 2, 4, 8, and 16mg/mL of glucose (D-glucose, ≥99.5%, St. Louis, MO, USA) for 24 h, then observed cell morphology by an inverted microscope. 100 µL of the medium was retained and 10 µL of CCK-8 (Abmole, Shanghai, China) was added to each well. The values of optical density (OD) 450 nm were detected by a microplate reader (Bio-Rad, Hercules, CA, USA) after incubation for 1 h at 37 °C in a 5% CO_2_ cell incubator.

### 2.6. Flow Cytometric Analyze Cell Cycle

The combination of propidium iodide and double-stranded DNA can produce fluorescence. The BPTCs at 70% confluency in 6-well plates were collected and fixed in 70% ethanol (*v*/*v*) overnight at 4 °C. Subsequently, the fixed cells were centrifuged at 1000× *g* for 5 min and rewashed with cold PBS. The cells were stained with 0.5 mL propidium iodide solution (BD, Franklin Lakes, NJ, USA) and incubated at room temperature for 30 min. The fluorescence at 488 nm was measured with a flow cytometry (BDVerse, Franklin Lakes, NJ, USA). A ModFit Tutorial LT 5.0 was used to analyze the data of cell cycle. The cells proliferation index (PI) was calculated as PI = (S + G2M)/(G0G1 + S + G2M) [26,27,28].

### 2.7. ATP Content Determination

For the detection of intracellular ATP content, cells treated with different concentrations of glucose in 6-well plates were collected, centrifuged at 4000 r/min for 10 min, and resuspended with PBS. The cells were broken up by ultrasonication to release intracellular components and centrifuged at 2000–3000 r/min for 20 min. The supernatant was collected and measured according to the instructions of the Bovine Adenosine Triphosphate (ATP) ELISA kit (KEAIB, Shanghai, China) and the units were expressed in nmol/L.

### 2.8. RNA Extraction and Quantitative Real-Time PCR

The total RNA from the BPTCs treated with different concentrations of glucose for 24 h was extracted and purified using the Steady Pure Universal RNA Extraction Kit (Accurate Biotechnology, Changsha, China) as per the manufacturer’s protocol. The concentration and purity of the RNA was measured with the NanoDrop ND-2000 UV-VIS spectrophotometer (Thermo Scientific, Waltham, MA, USA). The concentration of RNA was adjusted to be consistent and then reverse-transcribed to cDNA with the Toyobo reverse transcription kit (ReverTra Ace^®^ qPCR RT Master Mix, Servicebio, Wuhan, China) according to the manufacturer’s protocol. Quantitative real-time PCR (q-PCR) was performed with the FastFire qPCR PreMix (SYBR Green) kit (Vazyme, Nanjing, China). The *β-actin* was used as a housekeeping gene and the relative mRNA abundance of target genes were determined by the 2^−ΔΔ^Ct method [29]. The mean values of mRNA abundance in 1 mg/mL treatment were set to 1.00. The primer sequences sued in this study were listed in Table 1 (Sangon Biotech, Shanghai, China).

### 2.9. Western Blotting

The BPTCs treated with glucose with or without the addition of 100 nmol/L rapamycin for 24 h were collected for western blotting analysis. The BPTCs were washed three times with PBS, and 1 mL of cell RIPA solution was added to obtain total protein. The protein concentration was detected by the BCA protein concentration assay kit (Biosharp, Guangzhou, China). Western blot analysis was performed in brief as follows: 20 µg of protein/well was separated on 6–15% separating gel, 2–10% concentrating gel (Servicebio, Wuhan, China) and transferred to PVDF membrane (Absin, Shanghai, China). The membranes were sequentially closed with 5% skimmed milk generated in Tris-buffer, followed by incubation using primary antibodies, overnight at 4 °C. The membranes were incubated with horseradish peroxidase (HRP)-conjugated goat anti-rabbit IgG secondary antibody for 1 h at room temperature. Subsequently, the membranes were incubated with the specific ultrasensitive ECL chemiluminescent substrate (Biosharp, Guangzhou, China) and visualization of the proteins was achieved with the ChemiDOC MP (Bio-Rad). The mean values of protein in 1 mg/mL treatment were set to 1.00. Grayscale analysis was performed using ImageJ Software 1.8.0. The complete details of primary antibodies are listed in Table 2.

## 3. Statistical Analysis

All data in this study were statistically analyzed with one-way ANOVA followed by Duncan’s post hoc test using SPSS statistical software 27.0.1 (IBM Corp, Armonk, NY, USA). Data are expressed as means ± standard error of mean. *p* < 0.05 was considered statistically significance. Plots were drawn using GraphPad Prism 8.0 software.

## 4. Results

### 4.1. Identification of the BPTCs

**Figure 1:**
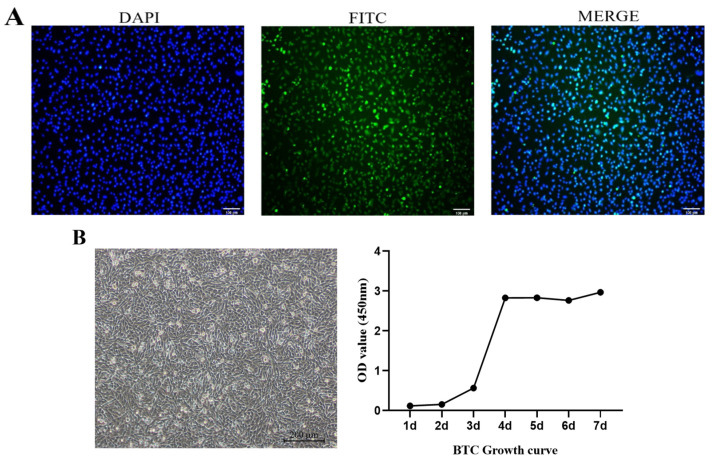
Identification of the BPTCs. (**A**) Immunofluorescence staining of cytokeratin 7 (200×; scale bar = 100 μm) in BPTCs using an inverted fluorescence microscope (Nikon TS100, Tokyo, Japan). FITC-stained cytokeratin 7 (green) and DAPI-stained nuclei (blue). (**B**) Growth curve of BPTCs.

The cell morphology showed a classic “pavement stone” shape through an inverted microscope. Immunofluorescence assays showed that the BPTCs marker CK7 protein was expressed positively (Figure 1A). The proliferation curve of the BPTCs was detected by the CCK8 method (Figure 1B), which showed that the cells were in a latent phase on the 2nd day, reached the logarithmic phase of cell growth on the 3rd day and the plateau phase on the 6th day, which was consistent with the cell growth pattern.

### 4.2. Effect of Glucose on Morphology, Viability and ATP Content of BPTCs

**Figure 2:**
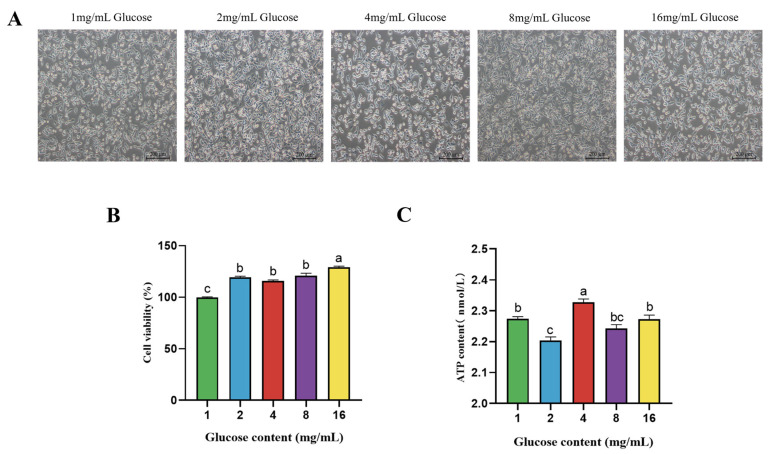
Effect of different glucose concentrations (1, 2, 4, 8 and 16 mg/mL) on the morphology (**A**), cell viability (**B**), and ATP content (**C**) of BPTCs after 24 h. The values are presented as mean ± SEM. Different superscripts (a–c) indicate significant differences.

The cell morphology and density were better at glucose treatment levels of 8 mg/mL compared to other treatment groups, as shown in Figure 2A. Furthermore, the cell viability of the BPTCs responded dose-dependent to glucose concentration; the cell viability was significantly higher at glucose concentration of 2, 4, 8 and 16 mg/mL than that at glucose concentration of 1 mg/mL (*p* < 0.05, Figure 2B). In addition, the ATP level at a glucose concentration of 4 mg/mL was significantly higher than that at 1 mg/mL (*p* = 0.0079).

### 4.3. Effect of Glucose on Cell Cycle of BPTCs

**Figure 3:**
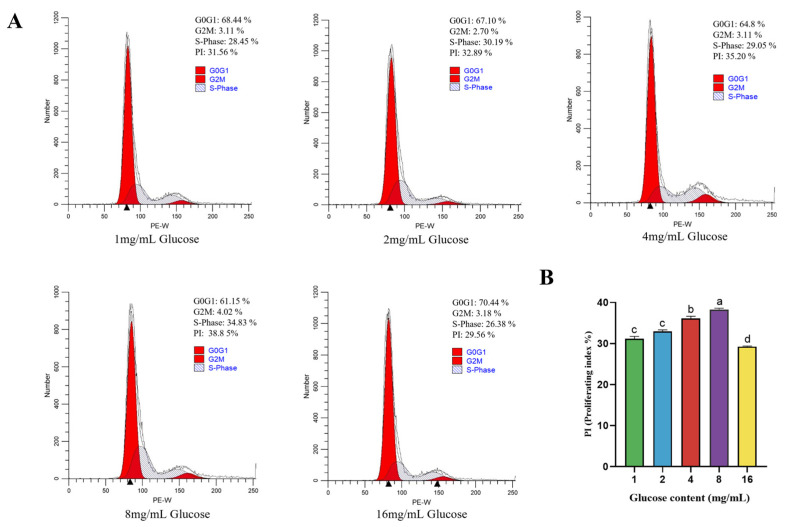
Effect of different glucose concentration (1, 2, 4, 8 and 16 mg/mL) on the cell cycle of BPTCs (**A**) and cells proliferation index (PI) (**B**). The values are presented as mean ± SEM. Different superscripts (a–d) indicate significant differences.

As shown in Figure 3, the S-phase value of the BPTCs increased with the glucose concentration increase and reached a maximum at 8 mg/mL (Figure 3A). The calculated values of the PI index also displayed a dose-dependent effect in the range of glucose concentration range of 1–8 mg/mL, with a maximum PI value of 38.85% at a glucose of 8 mg/mL (Figure 3B).

### 4.4. Effect of Glucose on Gene Expression of Transporter and Enzymes Related to Glucose Metabolism in BPTCs

**Figure 4:**
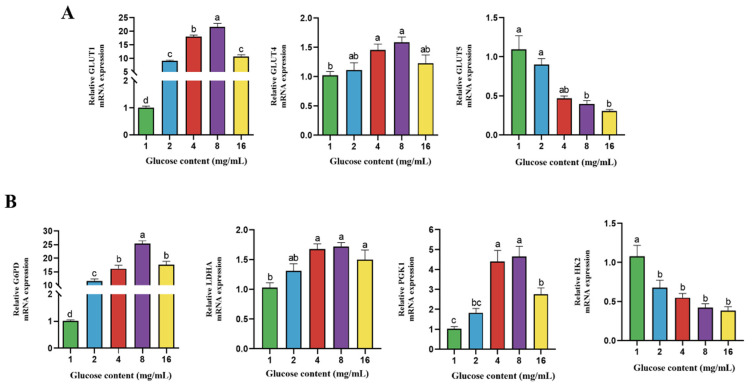
Effect of different glucose concentration (1, 2, 4, 8 and 16 mg/mL) on the gene expression of glucose transporter (**A**) and metabolism-related enzymes (**B**) in BPTCs. The values are presented as mean ± SEM. Different superscripts (a–d) indicate significant differences.

As shown in Figure 4, There was a dose-dependent effect of glucose on the gene expression of GLUT1 and GLUT4. Compared with 1 mg/mL glucose, the gene expression of GLUT1 was significantly upregulated at a glucose concentration of 2, 4, 8, and 16 mg/mL (*p* < 0.05); the expression of GLUT4 was significantly higher at a glucose concentration of 4 and 8 mg/mL than a glucose concentration of 1 mg/mL (*p* = 0.0354; *p* = 0.0037). The gene expression of GLUT5 was downregulated with the increase of the glucose concentration. There was a dose-dependent effect of glucose on the gene expression of G6PD and PGK1; a higher expression of G6PD, PGK1, and LDHA and a lower expression of HK2 was found at a glucose concentration of 4, 8, and 16 mg/mL when compared with glucose concentration of 1 mg/mL (*p* < 0.05).

### 4.5. Effect of Glucose on Gene Expression of PI3K-Akt/mTOR Signaling Pathway in BPTCs

**Figure 5:**
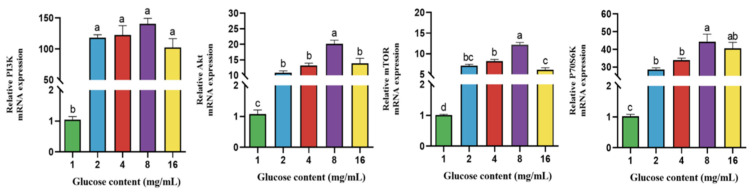
Effect of different glucose concentration (1, 2, 4, 8, and 16 mg/mL) on the gene expression of the PI3K-Akt/mTOR signaling pathway in BPTCs. The values are presented as mean ± SEM. Different superscripts (a–d) indicate significant differences.

As shown in Figure 5, the gene expression of PI3K, Akt, mTOR and P70S6K was significantly upregulated at a glucose concentration of 2, 4, 8, and 16 mg/mL than at a glucose concentration of 1 mg/mL (*p* < 0.05). The gene expression of Akt, mTOR, and P70S6K was significantly upregulated at a glucose concentration of 8 mg/mL than at a glucose concentration of 2 and 4 mg/mL (*p* < 0.05).

### 4.6. Effect of Rapamycin (Rapa) Decreased the Stimulation of Glucose Transporters in BPTCs

**Figure 6:**
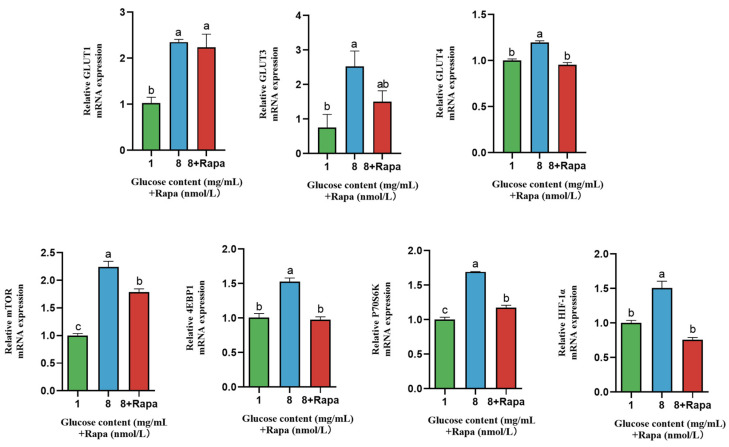
Effect of rapamycin and glucose on the protein expression of the mTOR signaling pathway in BPTC. The values are presented as mean ± SEM. Different superscripts (a–c) indicate significant differences.

As shown in Figure 6, the relative protein expression of mTOR, p-mTOR, P70S6K, p-P70S6K, 4EBP1, p-4EBP1, HIF-1α, and p-HIF-1α was significantly upregulated at a glucose concentration of 8 mg/mL than that at a glucose concentration of 1 mg/mL (*p* < 0.05), while Rapa downregulated the relative protein expression of mTOR, P70S6K, p-P70S6K, 4EBP1, p-4EBP1, and p-HIF-1α at glucose concentration of 8 mg/mL (*p* < 0.05).

**Figure 7:**
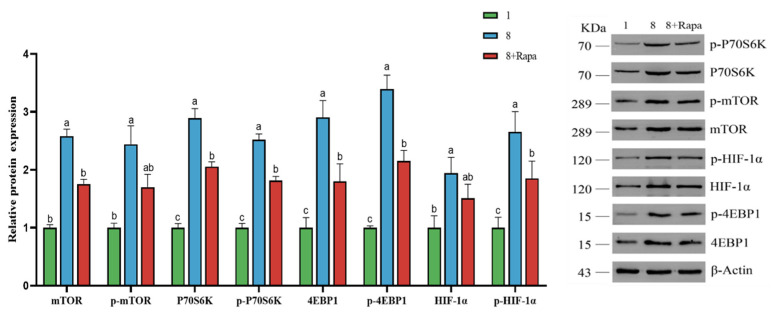
Effect of rapamycin and glucose on the gene expression of glucose transporters and the mTOR signaling pathway in BPTC. The values are presented as mean ± SEM. Different superscripts (a–c) indicate significant differences. The original images of the western blot are shown in the Supplementary Materials.

As shown in Figure 7, the gene expression of GLUT1, GLUT3, and GLUT4 was regulated at glucose concentration of 8 mg/mL than at a glucose concentration of 1 mg/mL (*p* < 0.05), while Rapa downregulated the relative gene expression of GLUT4 at a glucose concentration of 8 mg/mL (*p* < 0.05). The gene expression of mTOR, P70S6K, 4EBP1, and HIF-1*α* was regulated at a glucose concentration of 8 mg/mL than that at a glucose concentration of 1 mg/mL (*p* < 0.05), while Rapa downregulated the relative gene expression of mTOR, P70S6K, 4EBP1, and HIF-1*α* at a glucose concentration of 8 mg/mL (*p* < 0.05).

## 5. Discussion

The maintenance and activity of life in multicellular organisms depend on the availability of adequate nutrients, of which glucose has been shown to be a critical component in promoting cell proliferation [30,31,32]. Moreover, glucose is the most abundant nutrient transported by the placenta, meeting most of the energy needs of the fetus during pregnancy [33]. The restriction of glucose transportation in the placenta can limit fetal growth and development [34,35]. The placenta is the main link between the fetus and the mother, with functions such as digestion, excretion, respiratory, endocrine, and immunological [36]. The outer layer of the placenta is composed of trophoblasts, which play a crucial role in fetal development by coordinating all these functions [37]. In this study, the BPTCs were used as a model and found that glucose can promote cell proliferation. The gene expression levels of glucose transporters (GLUT1, GLUT3, and GLUT4) are lower at low glucose concentrations and significantly increase with an increasing glucose concentration, especially GLUT1. The research results indicate that compared to 1 mg/mL glucose, 8 mg/mL glucose promotes the transport of cellular nutrients. A study on goose liver cells found that glucose can regulate PI3K-Akt-mTOR activation to promote goose liver cell proliferation [38]. Consistent with this result, high dietary energy upregulated the gene expression of transporters in placental tissue [14], indicating that glucose transport in the placenta is affected by glucose concentration [39]. GLUT1 can efficiently transport glucose to the placental trophoblast ectoderm and subsequently to the placental vascular system to provide energy for the fetus in sows [21]. Since the glucose transported by the placenta is a key substrate for fetal oxidative metabolism, it is essential for maintaining the normal intrauterine development and survival of the fetus. Although GLUTs promote placental glucose uptake, excessively high concentrations of glucose may have negative effects. In this study, the gene expression of the GLUTs, glucose metabolism, and the mTOR pathway decreased at a glucose concentration of 16 mg/mL than that at glucose concentration of 8 mg/mL. Zhao et al. (2012) also reported the gene expression of GLUT1 downregulated in the 20 mM glucose treatment in primary bovine mammary epithelial cells (BMECs), compared with the 5 mM glucose treatment [40]. There is an effective counter-regulatory mechanism for the fetus to maintain normal glucose metabolism levels [41]. Due to the uncertainty in the function and the expression of transporters [42], there are limited reports on other glucose transporters in the placenta. In the current study, the expression of the classical glucose transporter (GLUT1 and GLUT4) increased with the increase of the glucose level, while the gene expression of GLUT5 was reversed. It is possible that GLUT5 is a high-affinity fructose transporter whose expression is primarily influenced by fructose and to a lesser extent glucose, yet the underlying molecular mechanisms of regulation by these sugars are largely unknown [43].

Glucose metabolism plays an important role in the metabolic processes of all organisms, especially mammals. Intracellularly, G6PD, LDHA, PGK1, and HKs are key enzymes in glucose metabolism, where G6PD and HKs are rate-limiting enzymes that regulate the glucose metabolism pathway [44]. In ruminants, four types of HKs have been identified that phosphorylate-absorbed glucose to glucose 6-phosphate, the first step in glycolysis occurring. Although glucose uptake by cells was regulated by HKs, the gene expression of HKs did not affect by glucose concentration in BMECs [40]. In this study, the gene expression of HK2 was downregulated at high glucose concentrations compared to 1 mg/mL glucose. Increased glucose levels stimulate glucose transport and this effect may act through a mechanism other than regulating the mRNA abundance of HK2, while HK2 plays a key role in regulating the glucose uptake by regulating glucose availability [40]. G6PD is the first rate-limiting enzyme in the pentose phosphate pathway, which is the main source of the reduced nicotinamide adenine dinucleotide phosphate (NADPH) production in mammals [45]. Previous studies have shown that 10 mM glucose resulted in significantly increased G6PD enzyme activity compared to 5 mM glucose in Atlantic salmon hepatocytes [46]. Furthermore, the optimal glucose concentration (5.6 mM) promoted the gene expression of G6PD in mature oocytes, as well as LDHA, thereby promoting embryonic development [47]. LDHA is an enzyme that catalyzes the conversion of pyruvate to lactic acid, which is the final step in glycolysis. LDHA regulated the protein expression of GLUT1 and basal glucose uptake in 3T3-L1 adipocytes [48]. Similarly, a high glucose concentration (4, 8, 16 mg/mL) increased the gene expression of LDHA compared with 1 mg/mL glucose in this study. PGK1 is a protein kinase that synergizes with mitochondrial function to produce the first ATP during glycolysis. In the current study, the ATP content and the gene expression of PGK1 in BPTCs were significantly higher in the 8 mg/mL glucose treatment than those in the 1 mg/mL glucose. These results indicated that the optimal glucose concentration (8 mg/mL) could promote the gene expression of glucose transporters and metabolic related enzymes, enhancing the energy supply efficiency in BPTCs. However, this study detected the gene expression of glucose metabolism enzymes by adding glucose to BPTCs. Considering the different physiological conditions in vitro and in vivo, further in vivo experiments are needed to deeply understand the complex mechanism of glucose transport and metabolism in placental tissue of cows during pregnancy.

It is well known that the animal organism has mechanisms that respond to changes in glucose concentration and maintain intracellular homeostasis. The main response pathway is the mTOR, which is directly involved in the synthesis of proteins and enzymes in the metabolic process after sensing changes in glucose concentration [49]. In placental tissue, this sensing element integrates maternal and fetal signals through trophoblast cells and continuously regulates placental function, thus ensuring optimal resource allocation between mother and fetus. To investigate whether glucose transport in BPTC mediated by the mTOR pathway, this study examined the effect of Rapa on BPTC at optimal glucose levels and analyzed the gene expression of GLTUs. In this study, the optimal glucose (8 mg/mL) treatment upregulated the expression of genes in the PI3K/Akt/mTOR signaling pathway. Subsequently, 100 nmol/L Rapa treatment decreased the gene and protein expression of mTOR, p-mTOR, P70S6K, p-P70S6K, 4EBP1, p-4EBP1, and HIF-α. Meanwhile, the gene expression of GLTU4 was significantly decreased by Rapa treatment. Downstream regulators of the mTOR pathway, such as 4EBP1 and P70S6K, are involved in protein synthesis to promote cell proliferation [38,50]. These findings of the study suggested that the mTOR signaling pathway sensed changes in glucose level, thereby controlling glucose uptake by regulating gene expression of GLUTs in BPTCs.

## 6. Conclusions

In summary, the results of this study suggested that cell activity and proliferation, glucose transporters, ATP content, and mTOR signaling pathways were affected by glucose levels in BPTCs. Besides, inhibition of the mTOR signaling pathway downregulated the gene expression of GLTU4 and the proteins expression in the mTOR signaling pathway after glucose addition. These findings indicated that glucose can affect the glucose transport by regulating the mTOR signaling pathway in BPTCs, which provided fundamental data on the regulatory mechanism of glucose transport in BPTCs. However, in vitro cell studies cannot completely represent the complex mechanisms of blood glucose in cows during pregnancy. Therefore, further in vivo experiments are necessary to understand the mechanism of glucose transport between mother and fetus through the placenta in cows during pregnancy.

## Figures and Tables

**Table 1:** Primer sequences for quantitative real-time PCR This is a table.

Gene Name	Forward (5′→3′)	Reverse (5′→3′)	Accession No.
*mTOR*	AAACCCAGGTGTGATCAATAATGTC	CATCAACCCATTTCCTCATTTCA	XM_002694043.6
*PI3K*	CCGGTTCCGCCAGTGTT	CCCATGCCGGCGTAAAA	NM_001206047.2
*Akt*	CCAGGTATTTTGATGAGGAGTTC	GTCTTGGTCAGGTGGCGTAA	NM_173986.2
*P70S6K*	TTTGCCTCCCTACCTCACG	GCCAGCAGTTCTTCCCAGTT	NM_205816.1
*G6PD*	CGCCTCAACAGCCACATA	CAGGTCCCTCCCAAACG	NM_001244135.2
*PGK1*	CATCCTGGGCGGAGCTAAAGTTG	GGTCCATTCCACACGATCTGCTTAG	NM_001034299.1
*LDHA*	TTGGTCCAGCGTAACGTGAACATC	ACTCCACTCCATACAGGCACACTAG	NM_174099.2
*HK2*	GGAGATTGCTAAGCGTTTTCG	AAGCCGTAGGGTGAGTGGTG	XM_015473383.2
*GLUTI*	TGGGCTTCTCAAAACTGGG	GGATGCCGACGACGATG	NM_174602.2
*GLUT3*	CGGCAACCCATCATTATCTC	CTGGACACCCGCATCTTT	NM_174603.3
*GLUT4*	AGGAGGAGAAGCGGAAGC	AATGGCGATGACGAGGG	NM_174604.1
*GLUT5*	CGTGGTGGAACTAATGGGG	CAAGCGGTGAAACAGACAGAG	NM_001101042.2
*β-actin*	TCCCTGGAGAAGAGCTACGA	TCCCTGGAGAAGAGCTACGA	NM_173979.3

*mTOR, Mammalian target of rapamycin; PI3K, Phosphatidylinositol3-kinase; Akt, Protein Kinase B, PKB; P70S6K, Ribosome S6 protein kinase; G6PD, Glucose-6-phosphate dehydrogenease; PGK1, Phosphoglycerate Kinase 1; LDHA, Lactate dehydrogenase; HK2, Hexokinase 2; GLUT1, GLUT3, GLUT4, GLUT5, Glucose transporter 1, 3, 4, 5; β-actin, beta-Actin.*

**Table 2:** Primary antibodies used for western blotting.

Antibody	Dilution Ratio	Source	Manufacturer
mTOR	1:1000	Rabbit	Affinity
p-mTOR	1:1000	Rabbit	Affinity
P70S6K	1:1000	Rabbit	ABclonal
p-P70S6K	1:1000	Rabbit	ABclonal
4EBP1	1:500	Rabbit	ABclonal
p-4EBP1	1:1000	Rabbit	ABclonal
HIF-1α	1:1000	Rabbit	ABclonal
p-HIF-1α	1:1000	Rabbit	ABclonal
β-actin	1:2000	Rabbit	Affinity
Goat Anti-rabbit IgG	1:5000	Goat	Affinity

mTOR, mammalian target of rapamycin; p-mTOR, phosphorylated mammalian target of rapamycin; P70S6K, 70 kDa ribosomal protein S6 kinase 2; p-P70S6K, phosphorylated 70 kDa ribosomal protein S6 kinase 2; 4EBP1, eIF4E-bind-ing protein 1; p-4EBP1, eIF4E-binding protein phosphorylation; HIF-1α, hypoxia-inducible factor 1-alpha; p-HIF-1α, phosphorylated hypoxia-inducible factor 1-alpha; β-actin, beta-actin.

## Data Availability

The data presented in this study are available on request from the corresponding author. The data are not publicly available due to their containing information that could compromise the privacy of research participants.

## Supplementary Materials

The following supporting information can be downloaded at: https://www.mdpi.com/article/10.3390/ani14010040/s1, the original images of the western blot.
