# Supplementary figures and images for: Glucose Regulates Glucose Transport and Metabolism via mTOR Signaling Pathway in Bovine Placental Trophoblast Cells
Source: Animals (Basel). 2023 Dec 21;14(1):40. doi: 10.3390/ani14010040 (PMC10778405; doi:10.3390/ani14010040)

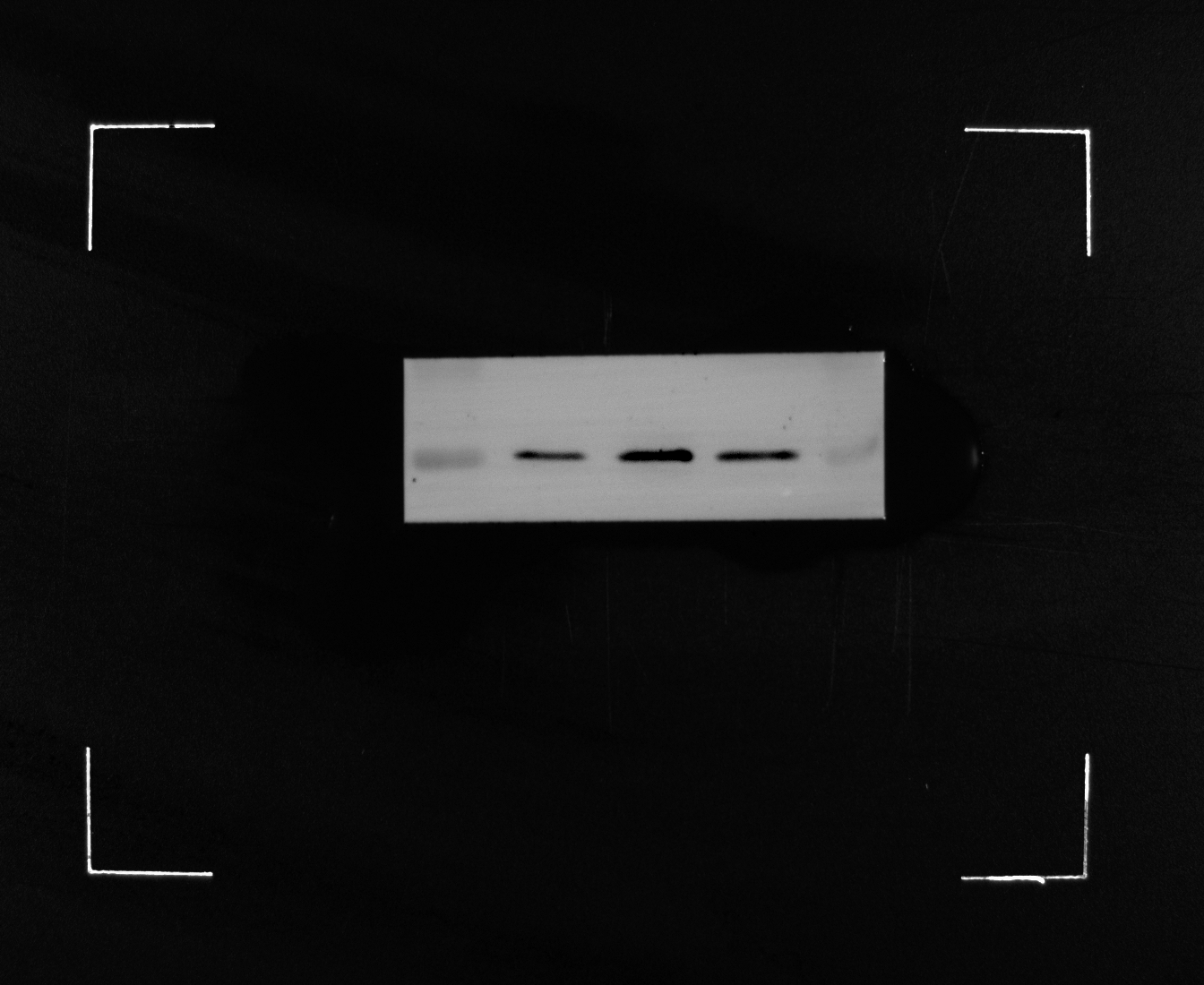

Supplement: Supplementary file 1 [file animals-14-00040-s001.zip › Westernblot/TIFF images/4EBP1-1.tif]

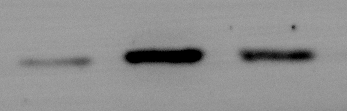

Supplement: Supplementary file 1 [file animals-14-00040-s001.zip › Westernblot/TIFF images/4EBP1-2.tif]

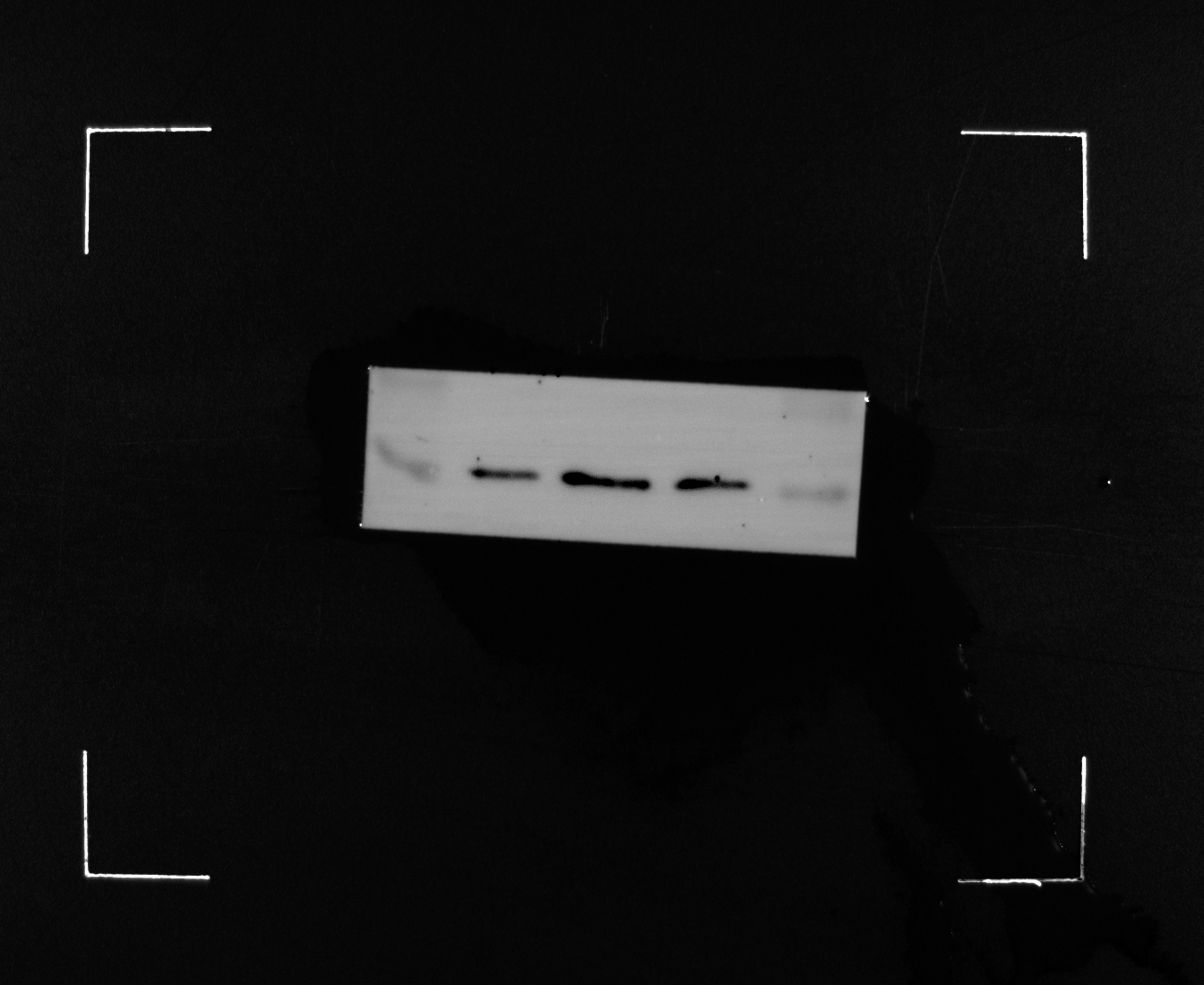

Supplement: Supplementary file 1 [file animals-14-00040-s001.zip › Westernblot/TIFF images/4EBP1-3.tif]

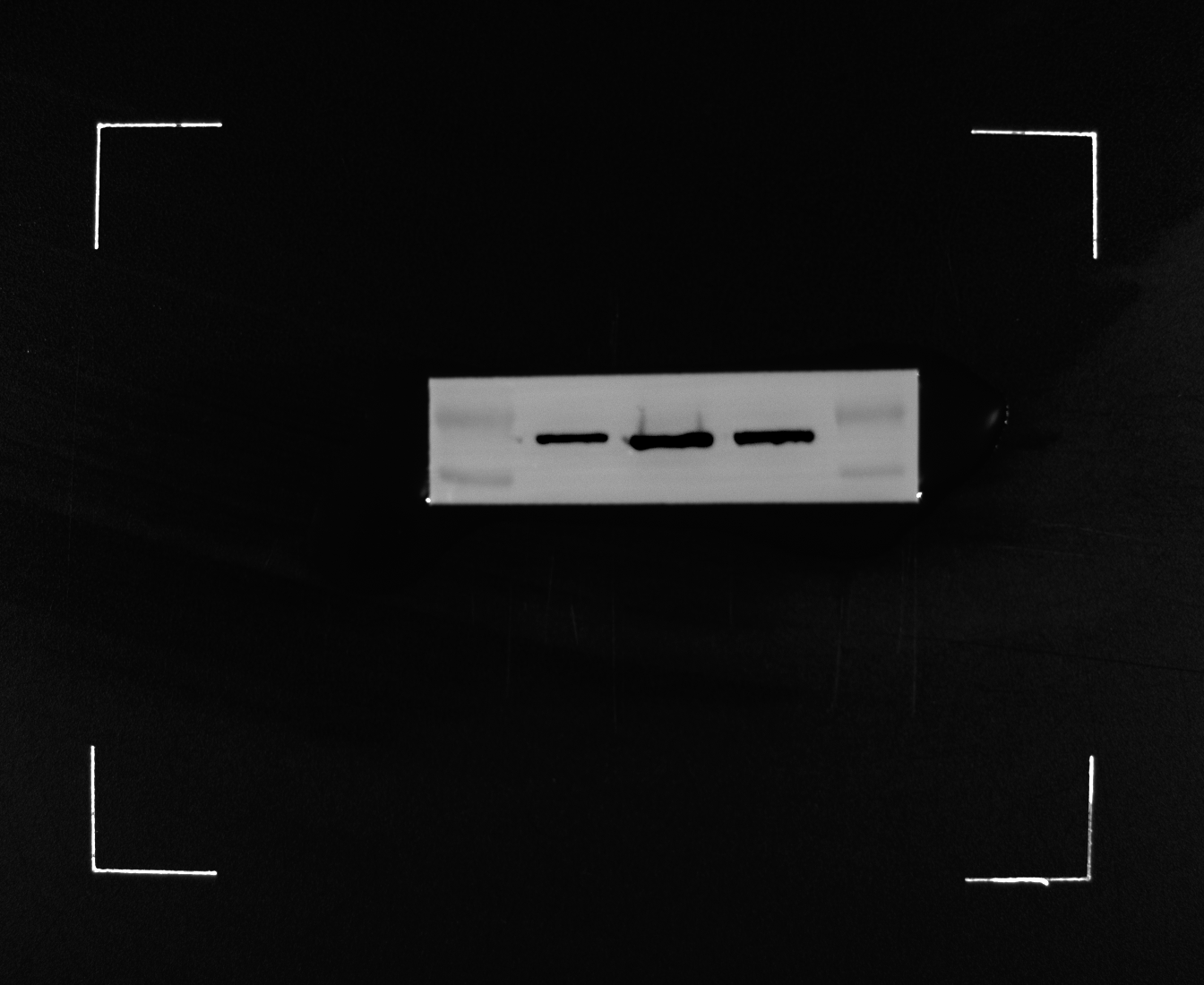

Supplement: Supplementary file 1 [file animals-14-00040-s001.zip › Westernblot/TIFF images/AMPK-1.tif]

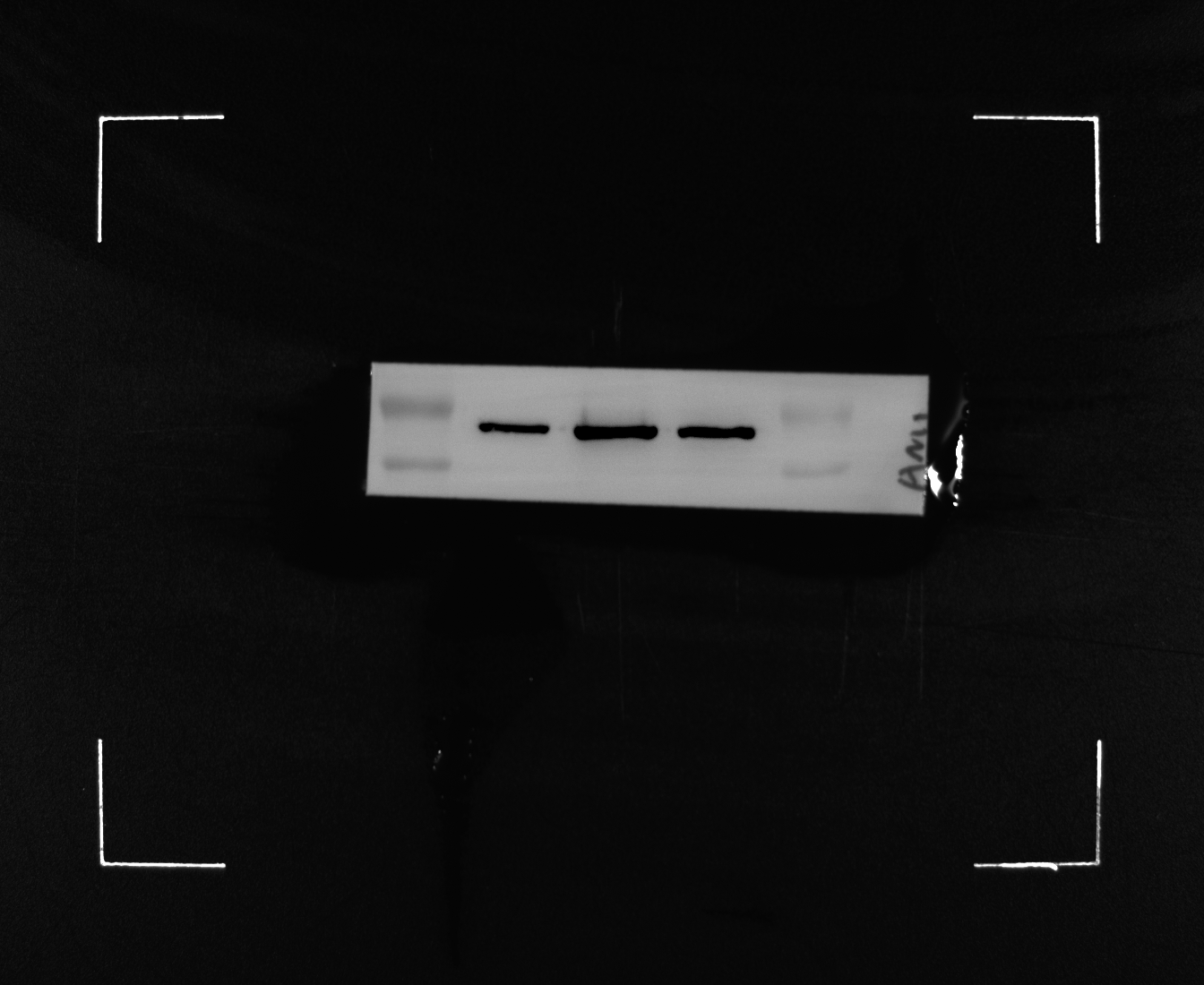

Supplement: Supplementary file 1 [file animals-14-00040-s001.zip › Westernblot/TIFF images/AMPK-2.tif]

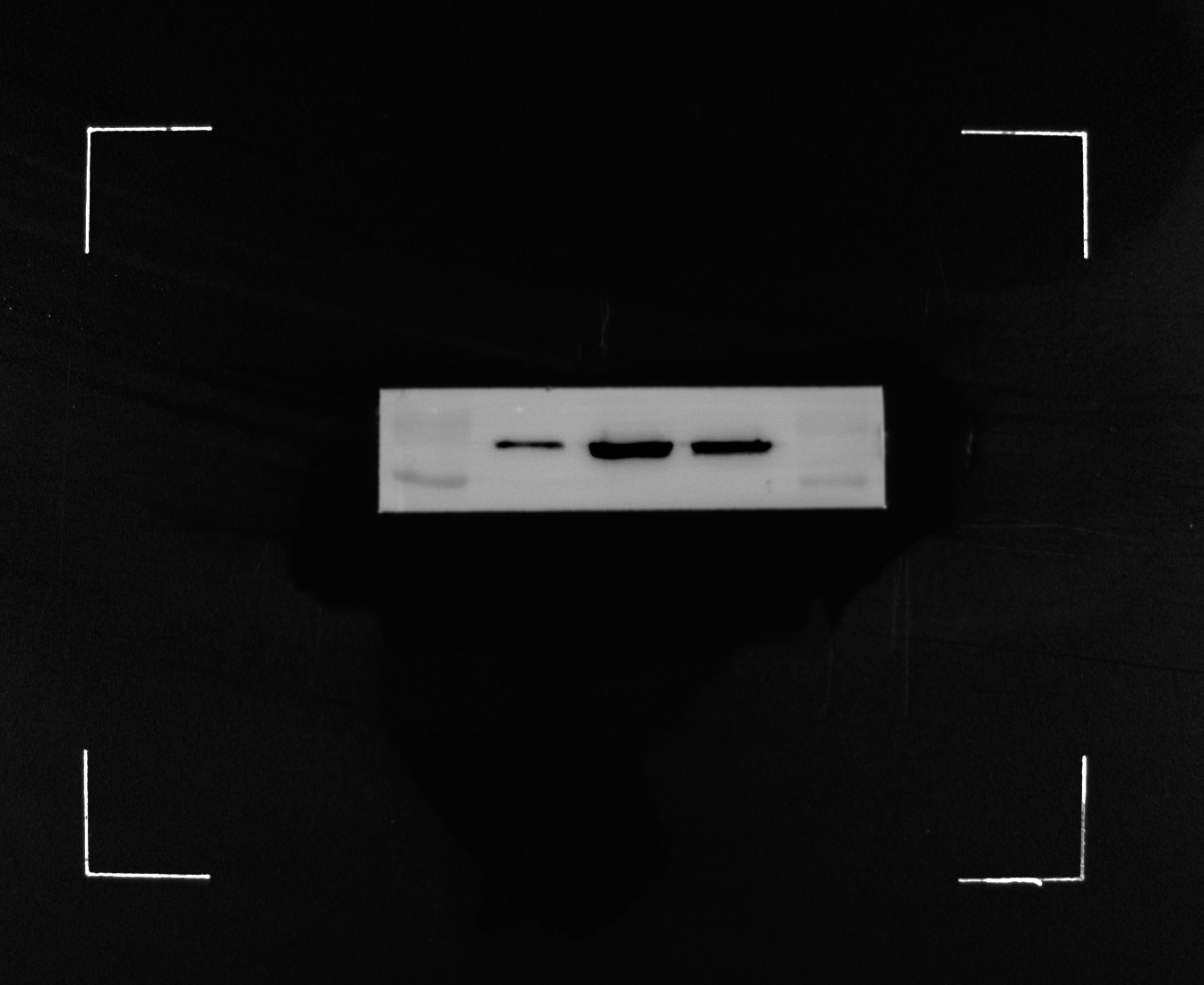

Supplement: Supplementary file 1 [file animals-14-00040-s001.zip › Westernblot/TIFF images/AMPK-3.tif]

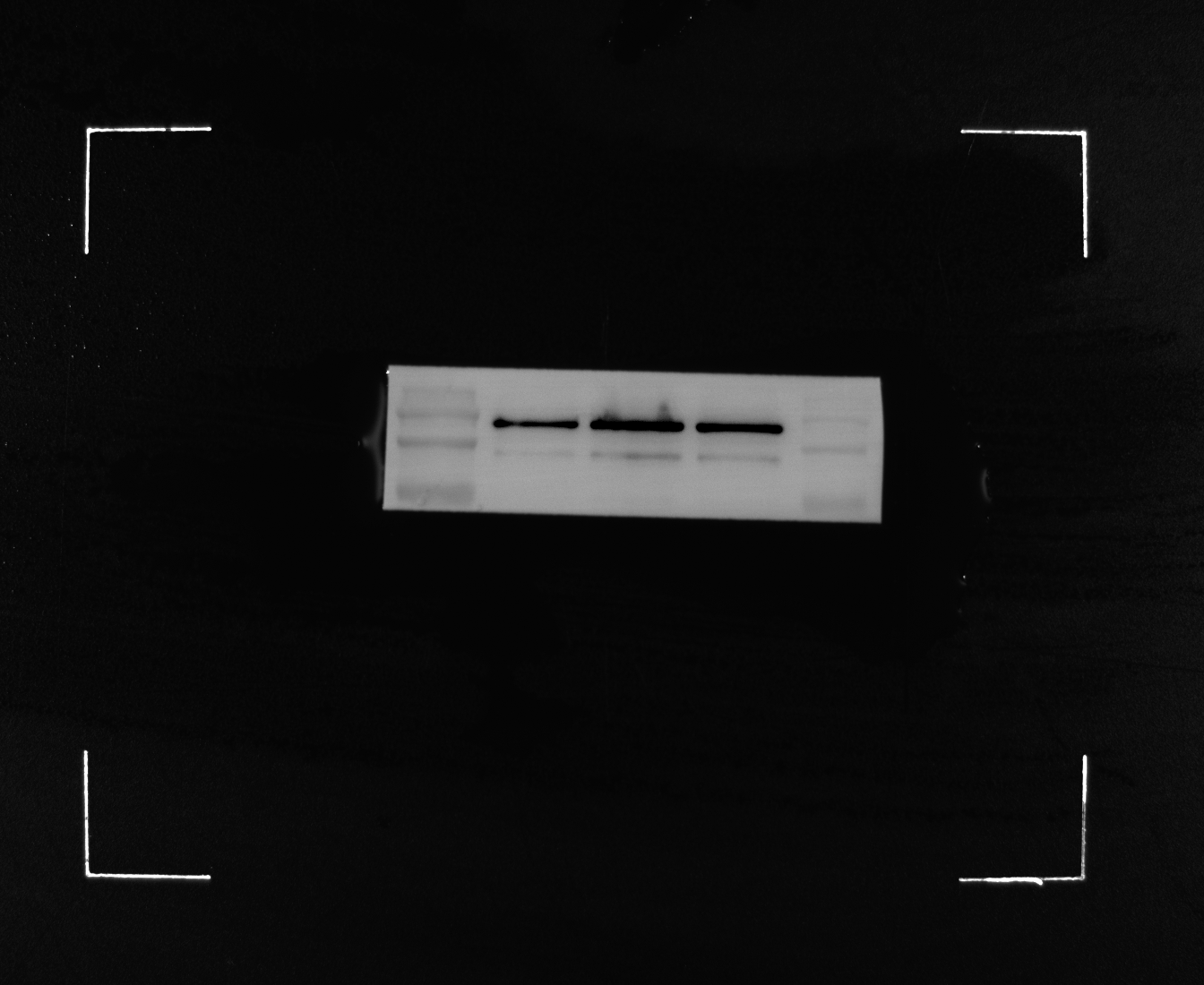

Supplement: Supplementary file 1 [file animals-14-00040-s001.zip › Westernblot/TIFF images/HIF-1a┴-1.tif]

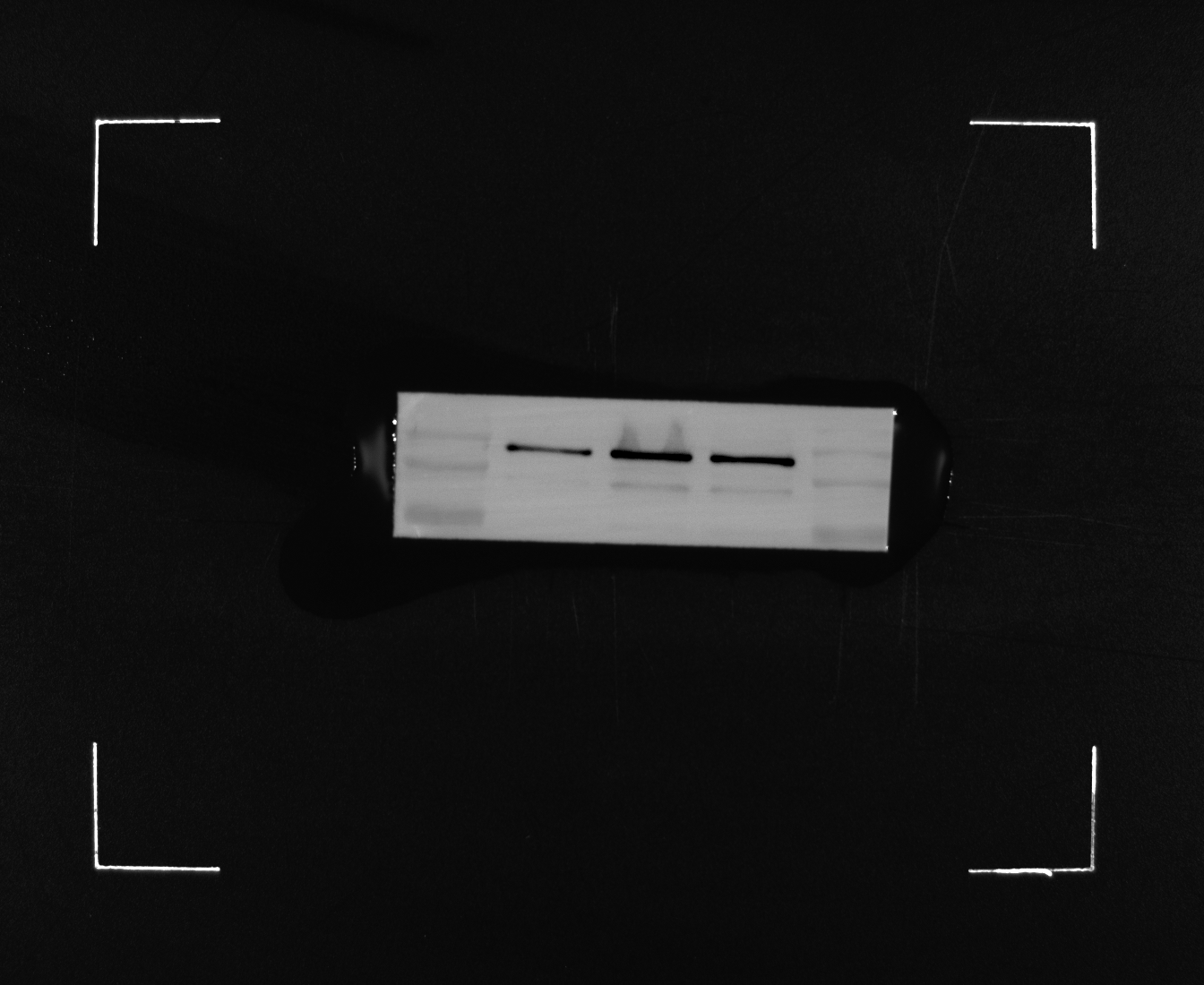

Supplement: Supplementary file 1 [file animals-14-00040-s001.zip › Westernblot/TIFF images/HIF-1a┴-2.tif]

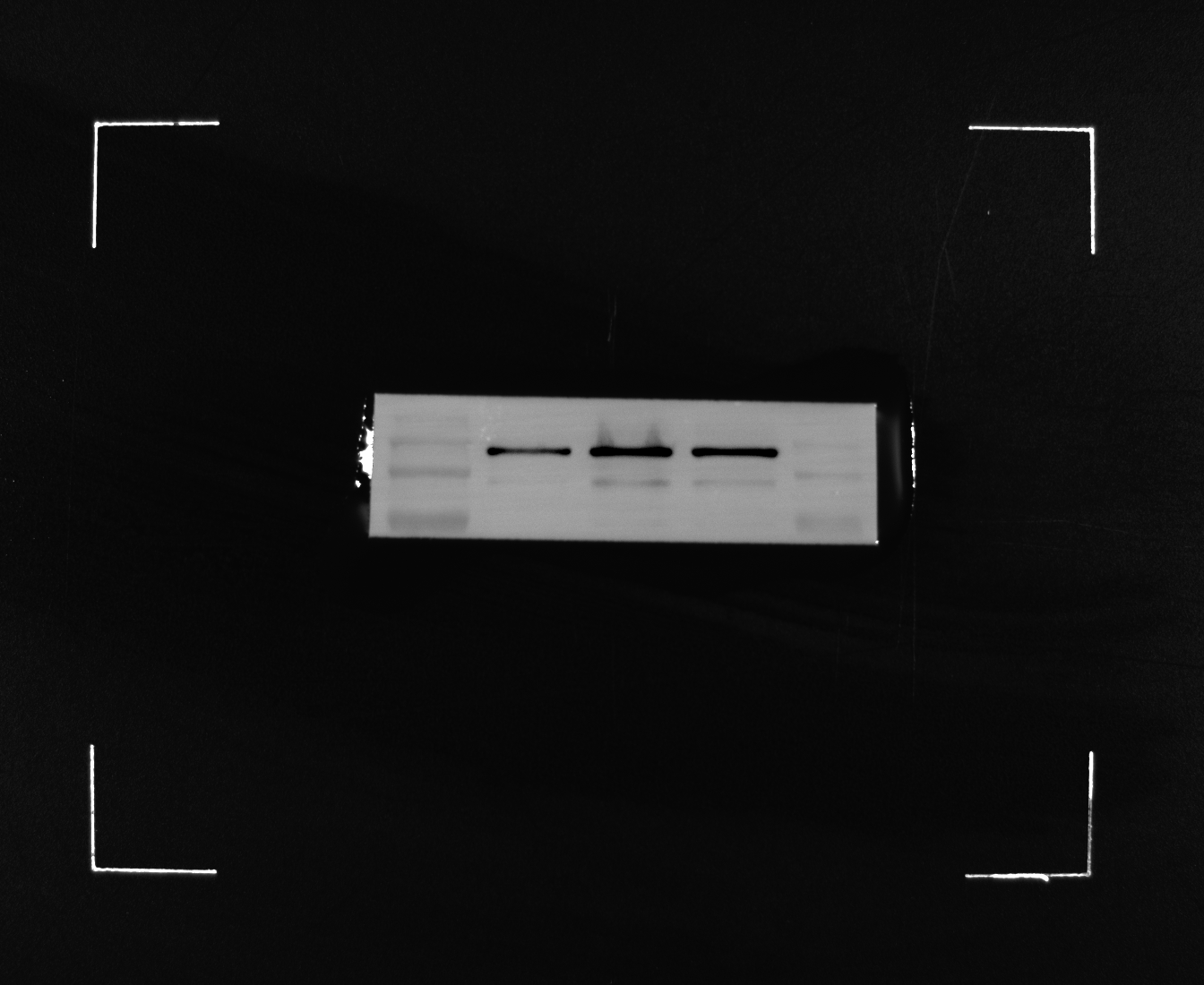

Supplement: Supplementary file 1 [file animals-14-00040-s001.zip › Westernblot/TIFF images/HIF-1a┴-3.tif]

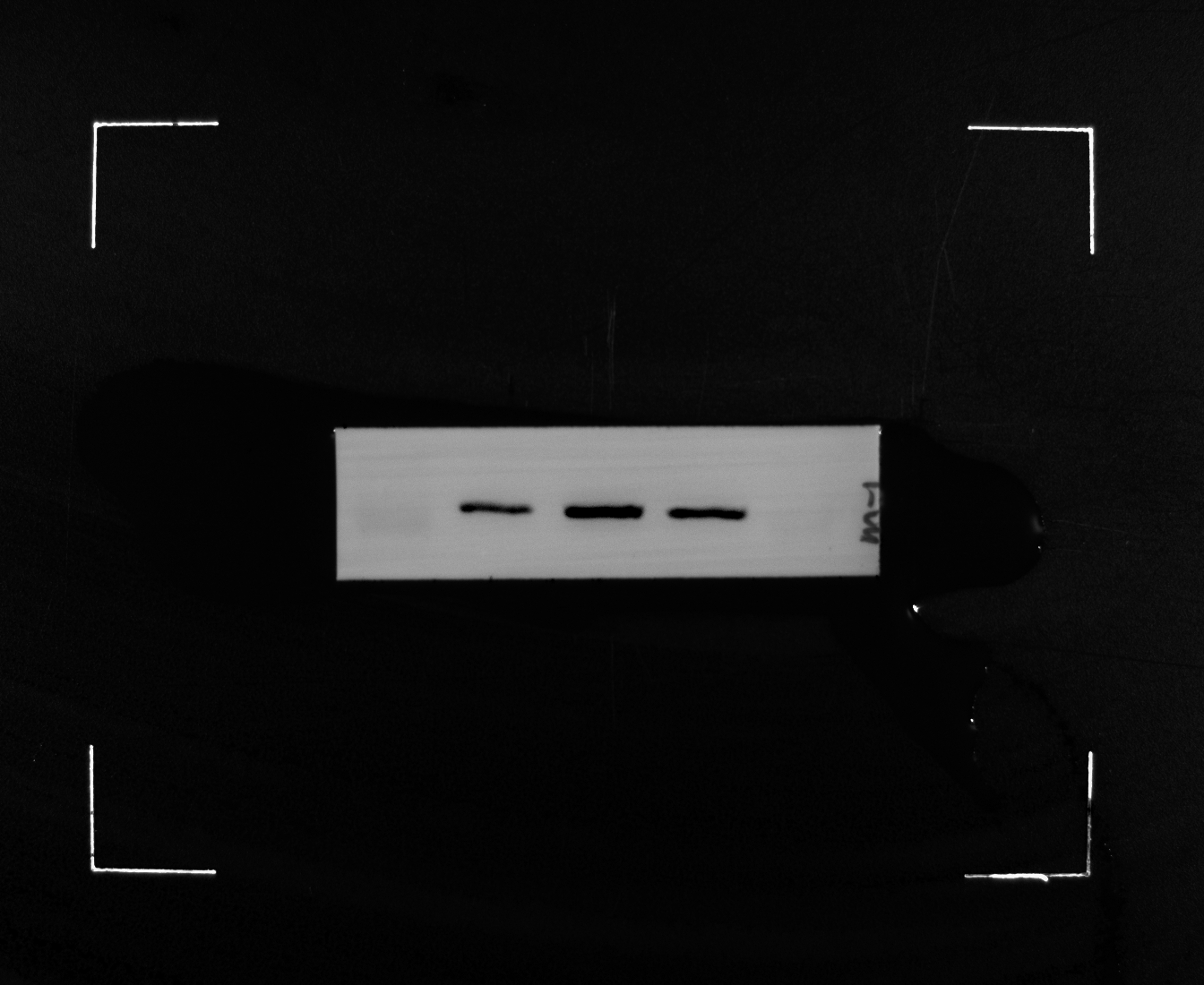

Supplement: Supplementary file 1 [file animals-14-00040-s001.zip › Westernblot/TIFF images/mTOR-1.tif]

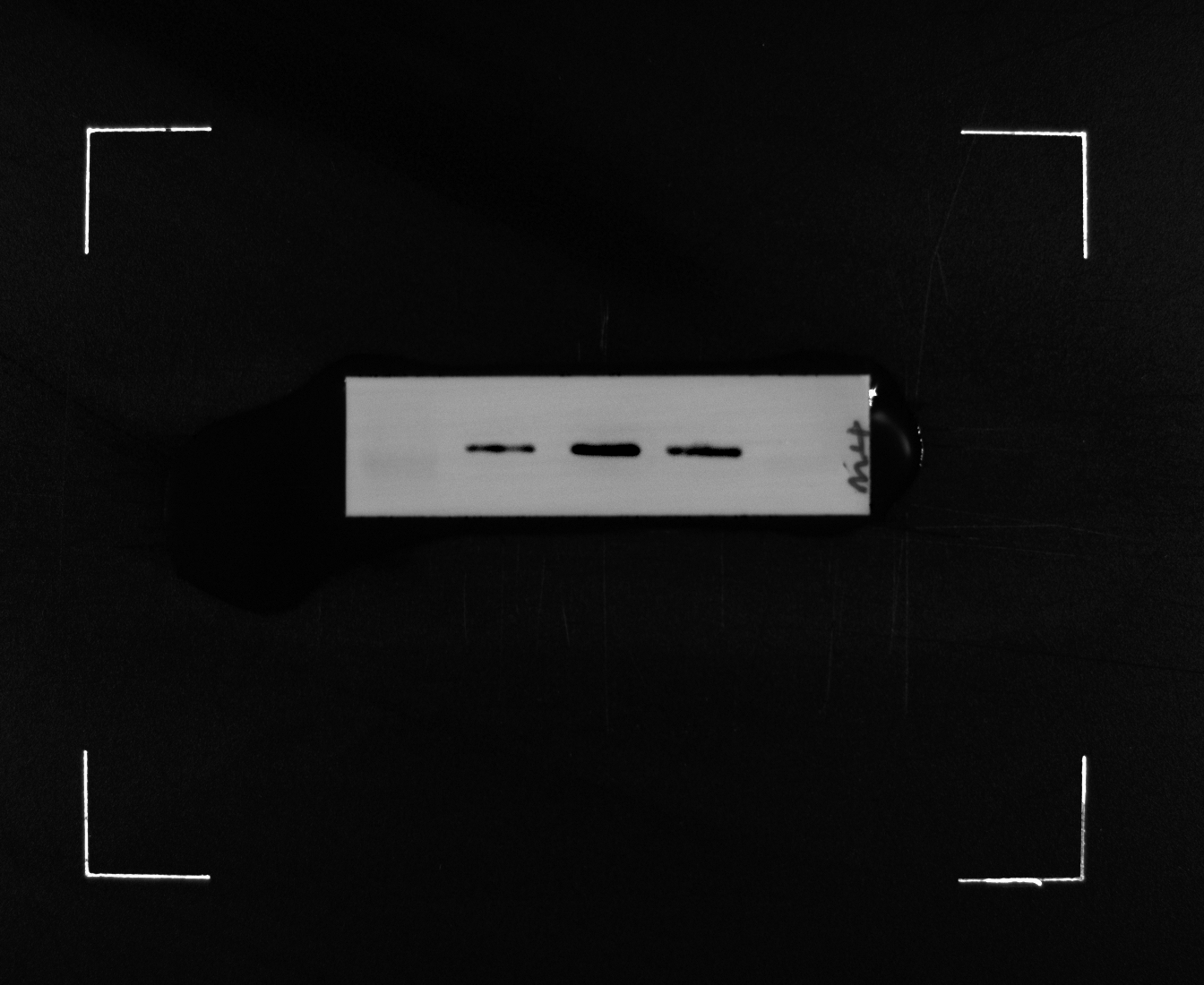

Supplement: Supplementary file 1 [file animals-14-00040-s001.zip › Westernblot/TIFF images/mTOR-2.tif]

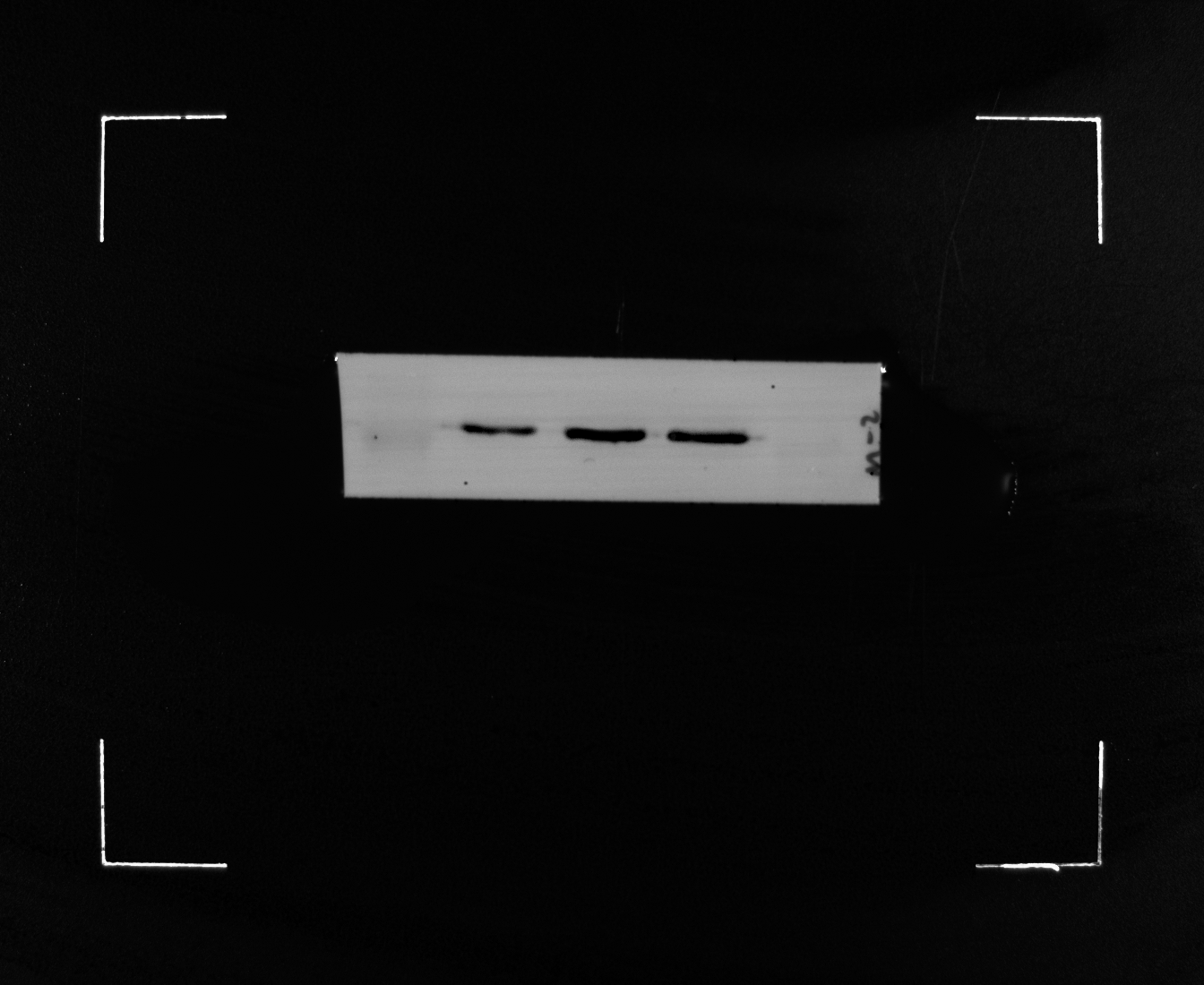

Supplement: Supplementary file 1 [file animals-14-00040-s001.zip › Westernblot/TIFF images/mTOR-3.tif]

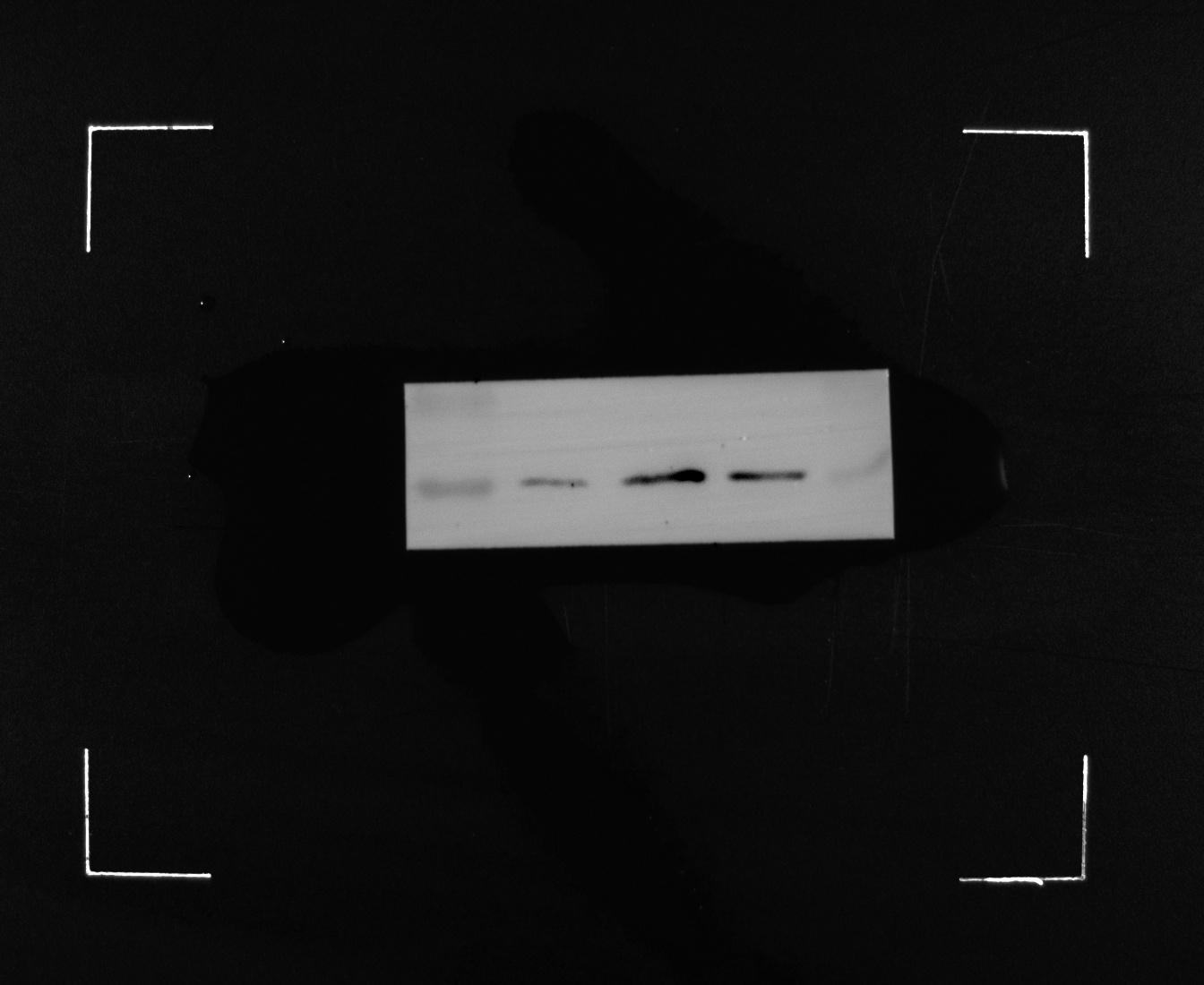

Supplement: Supplementary file 1 [file animals-14-00040-s001.zip › Westernblot/TIFF images/p-4EBP1-1.tif]

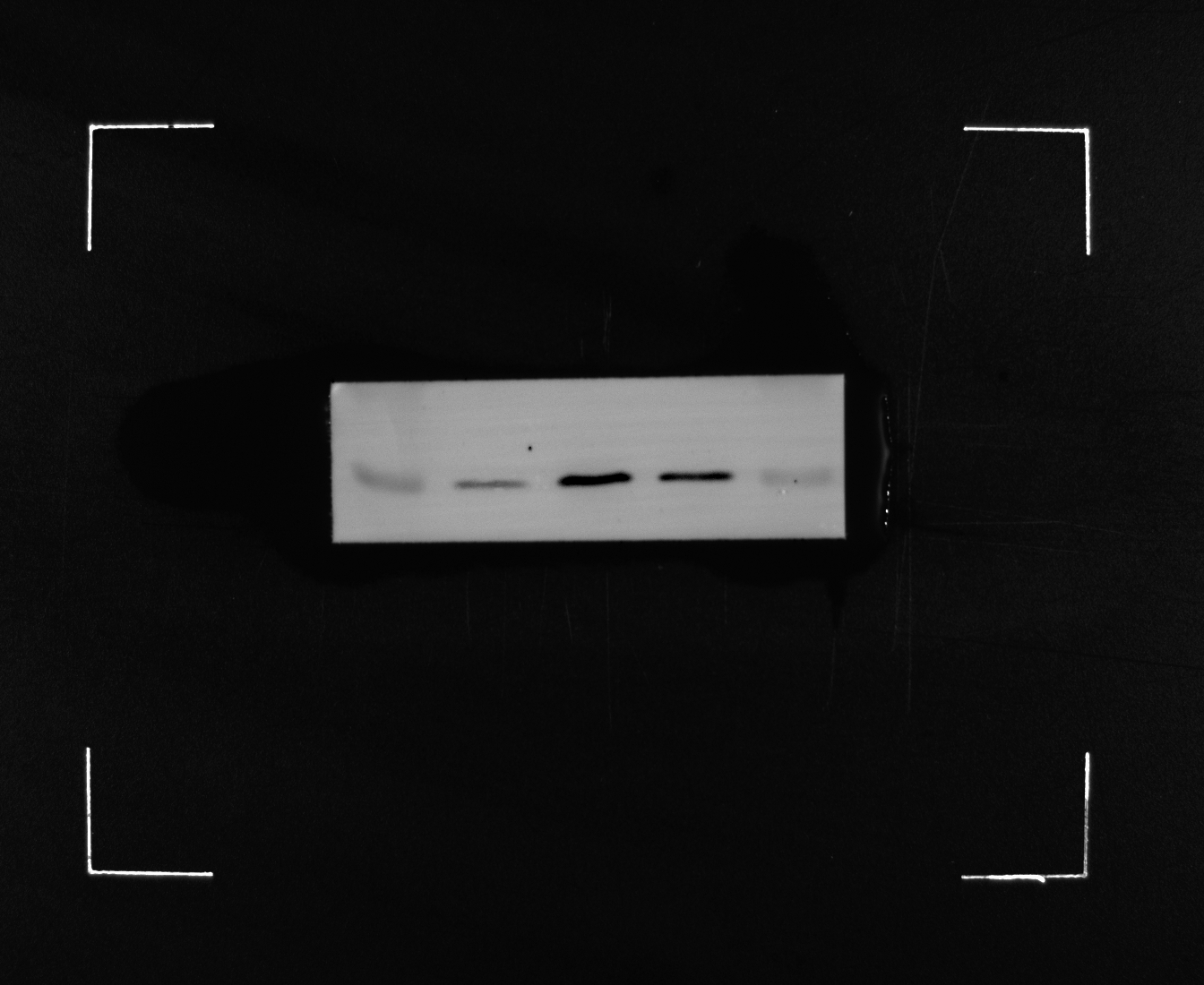

Supplement: Supplementary file 1 [file animals-14-00040-s001.zip › Westernblot/TIFF images/p-4EBP1-2.tif]

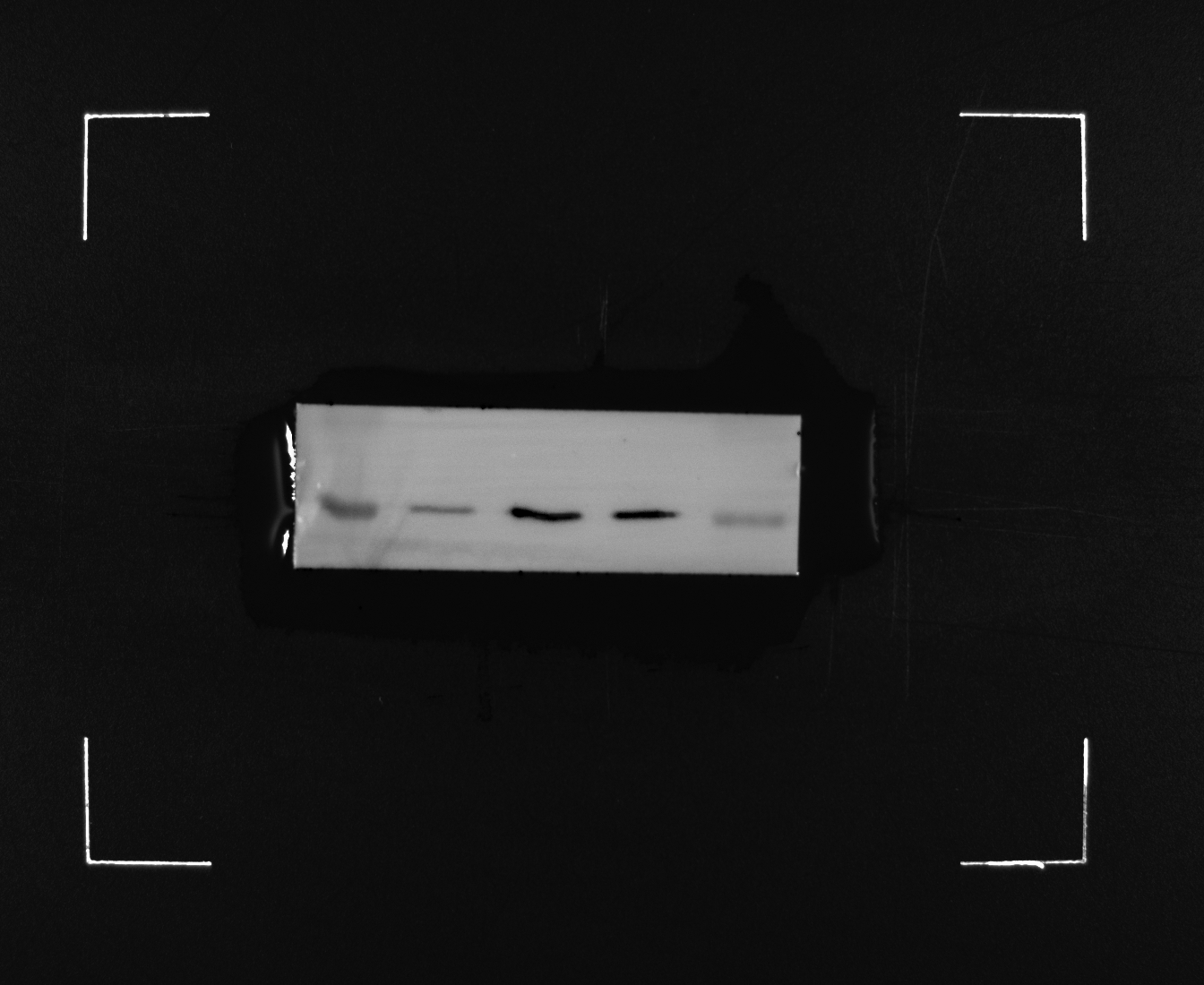

Supplement: Supplementary file 1 [file animals-14-00040-s001.zip › Westernblot/TIFF images/p-4EBP1-3.tif]

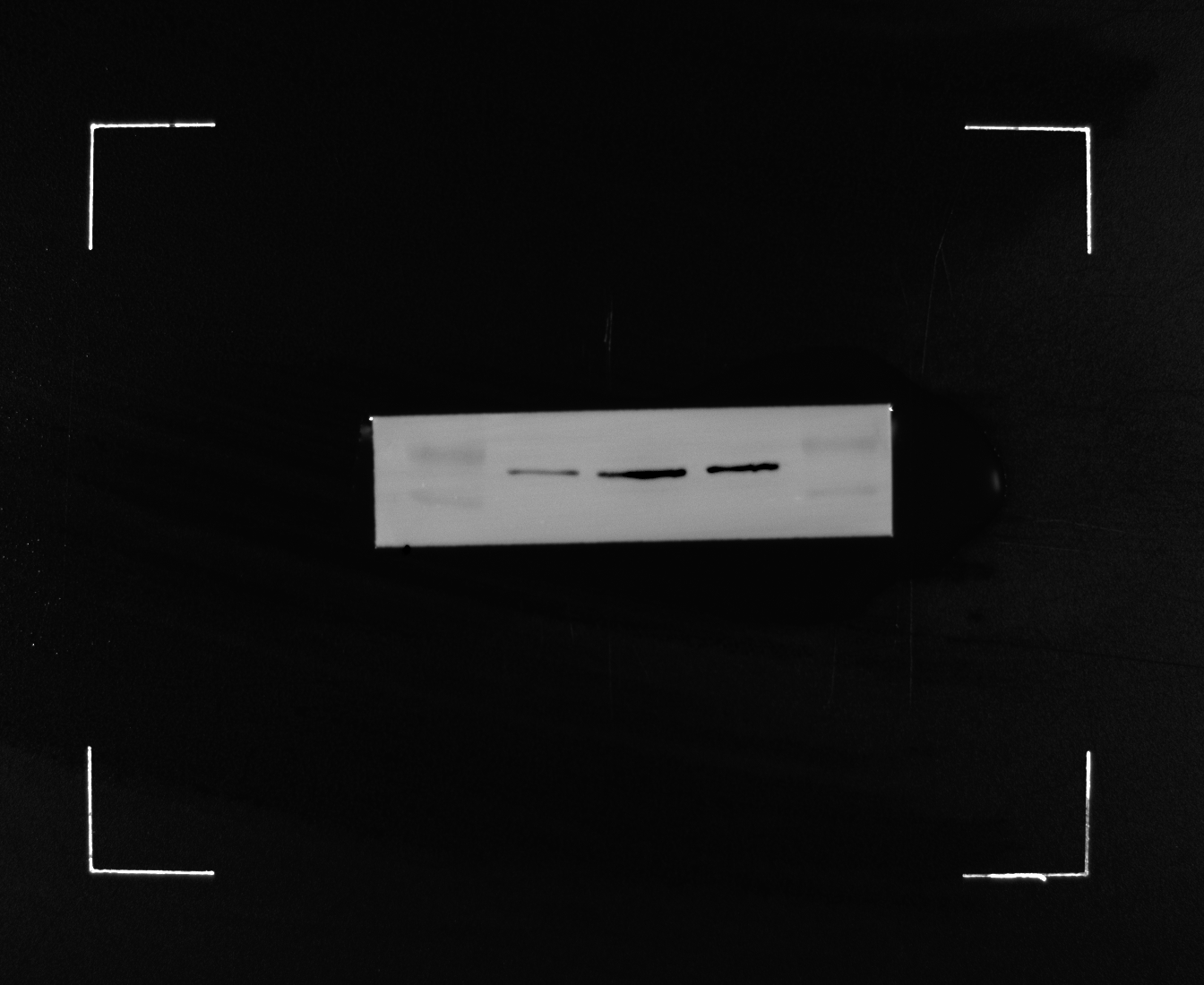

Supplement: Supplementary file 1 [file animals-14-00040-s001.zip › Westernblot/TIFF images/p-AMPK-1.tif]

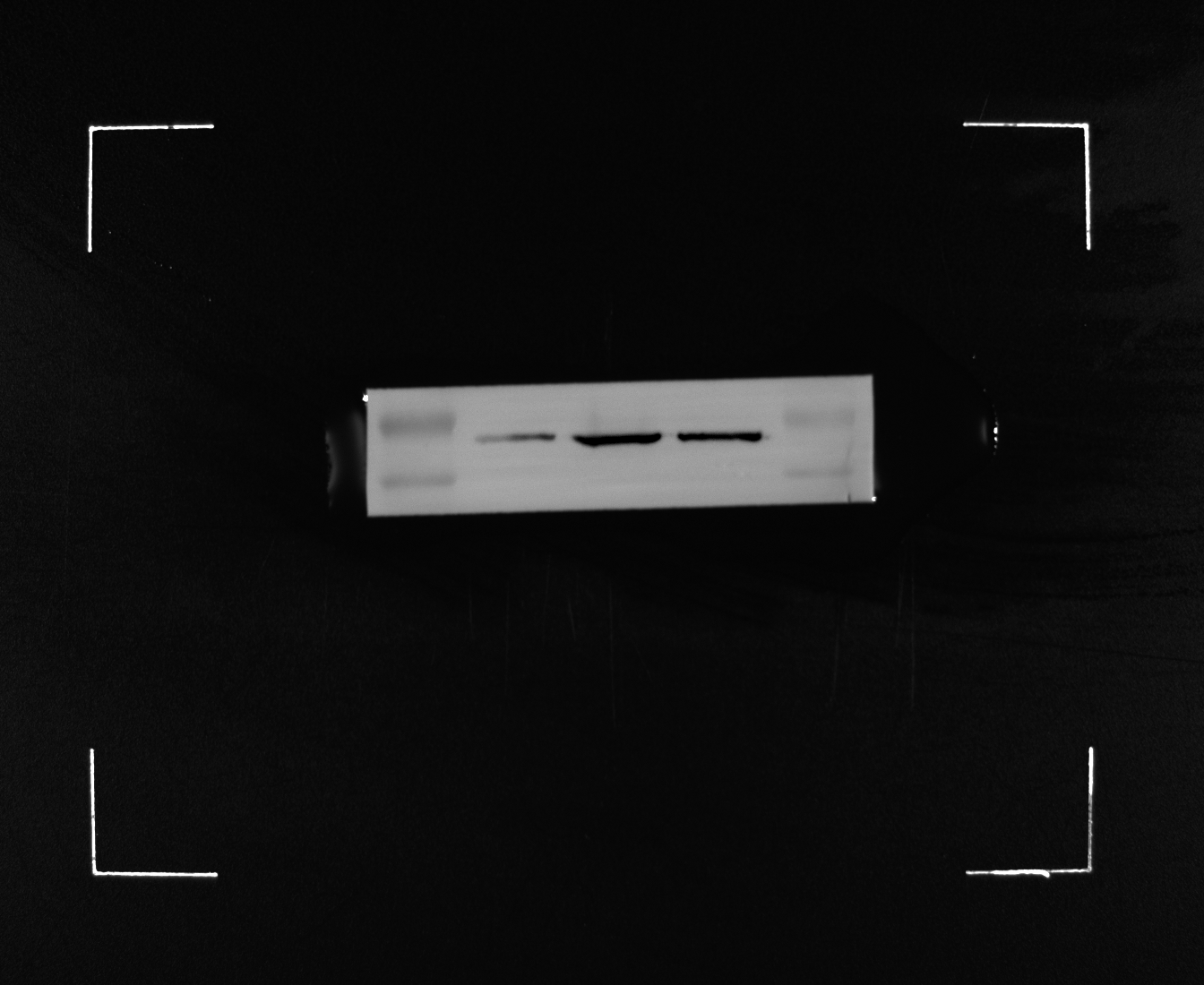

Supplement: Supplementary file 1 [file animals-14-00040-s001.zip › Westernblot/TIFF images/p-AMPK-2.tif]

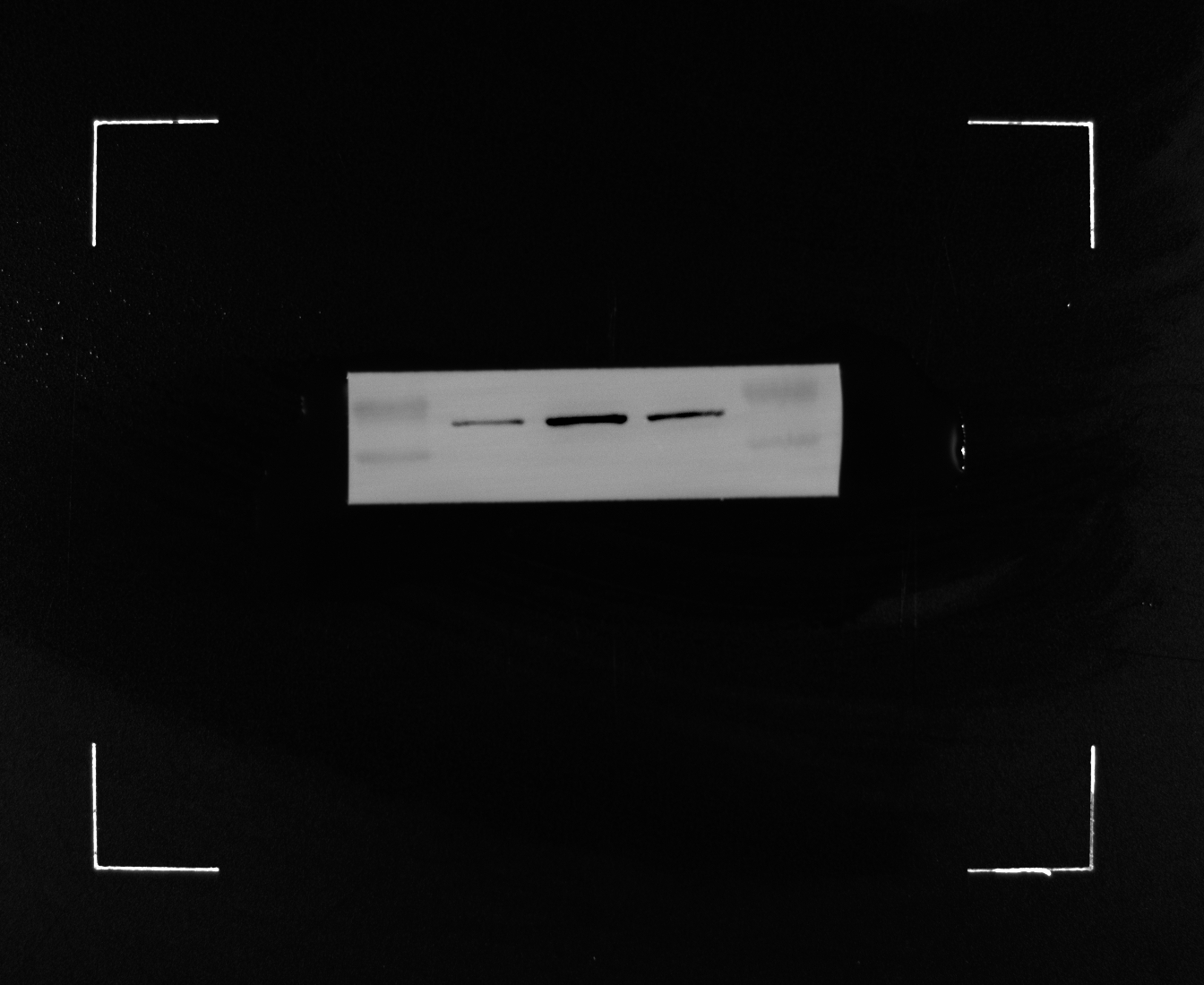

Supplement: Supplementary file 1 [file animals-14-00040-s001.zip › Westernblot/TIFF images/p-AMPK-3.tif]

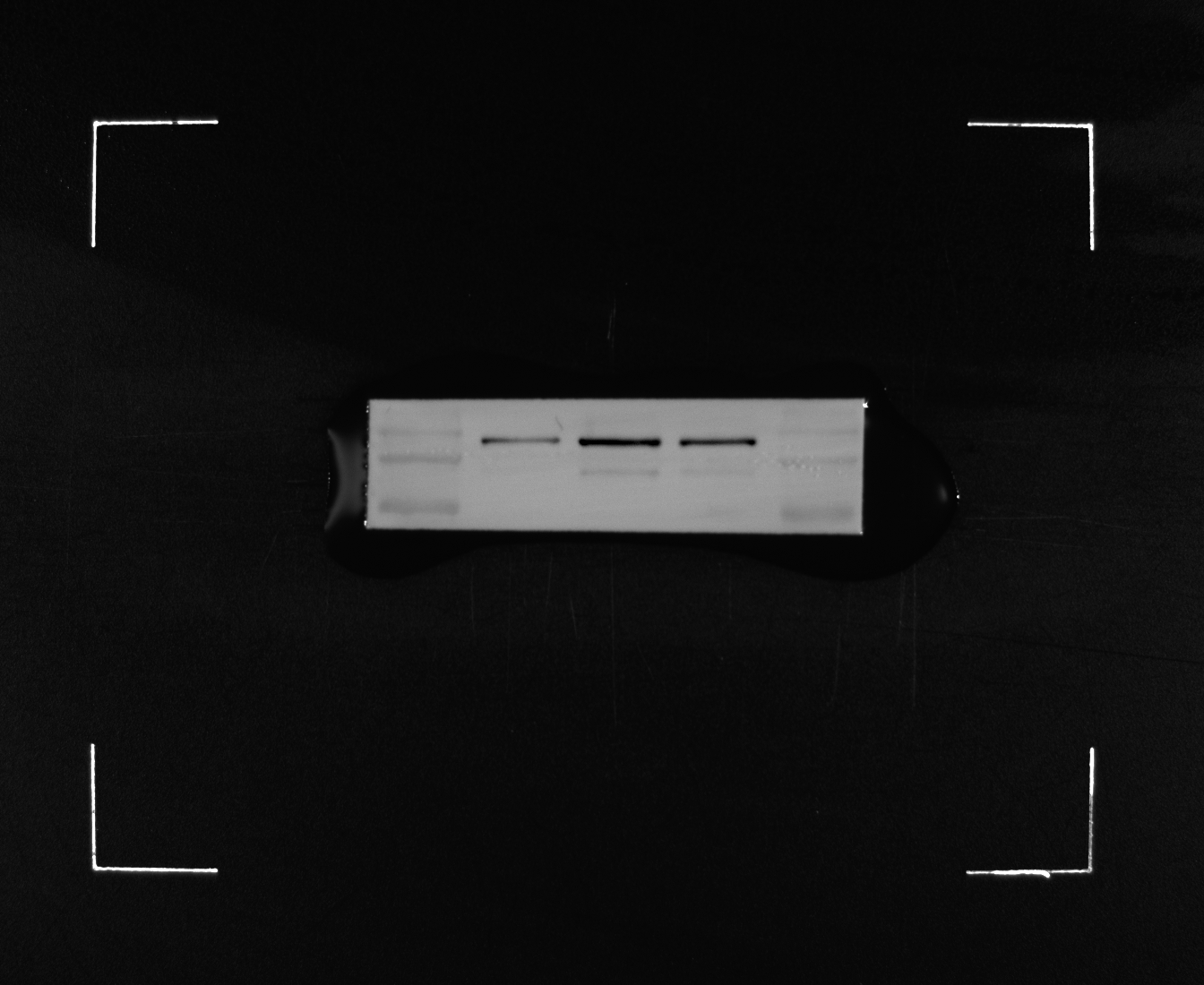

Supplement: Supplementary file 1 [file animals-14-00040-s001.zip › Westernblot/TIFF images/p-HIF-1a┴-1.tif]

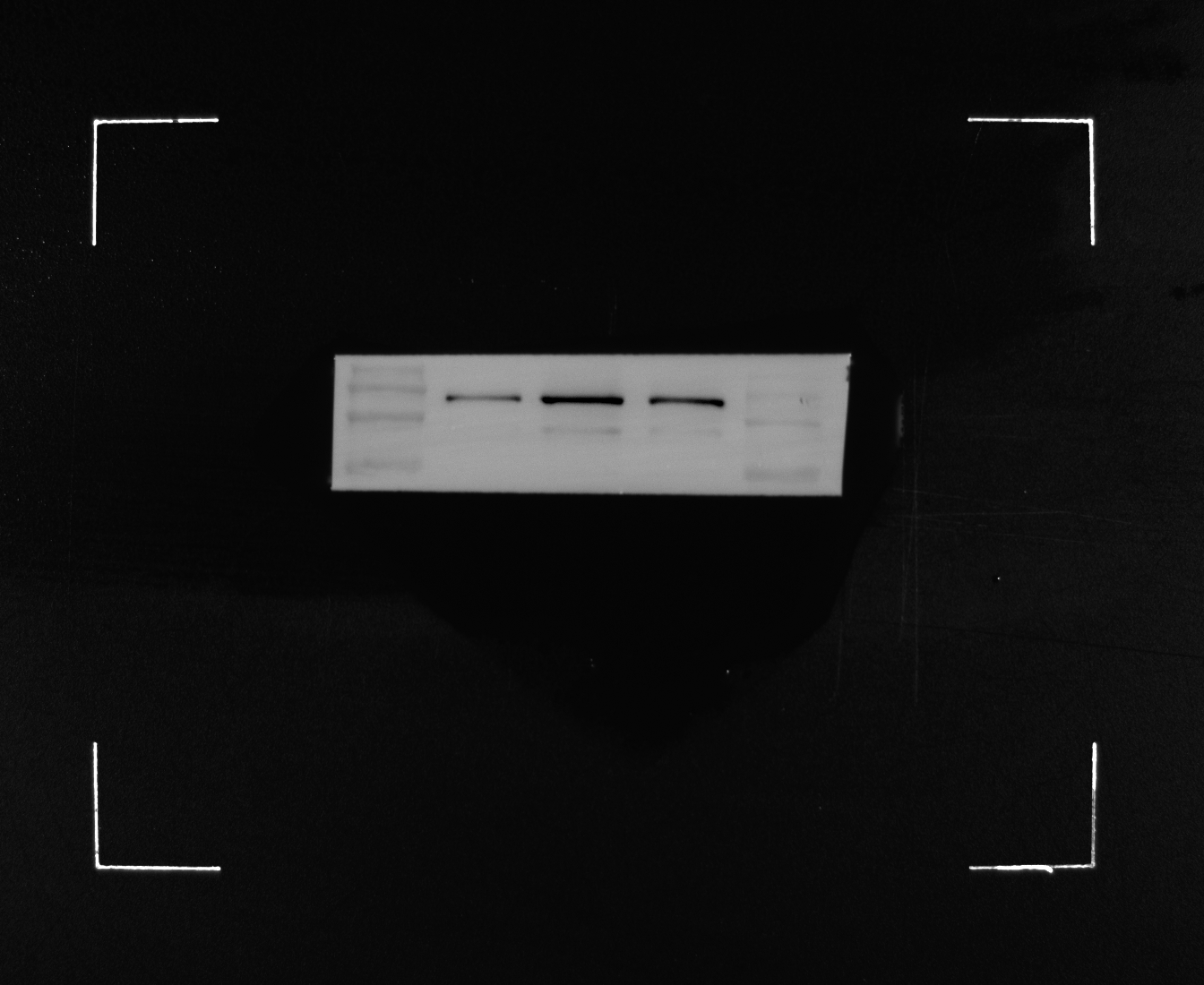

Supplement: Supplementary file 1 [file animals-14-00040-s001.zip › Westernblot/TIFF images/p-HIF-1a┴-2.tif]

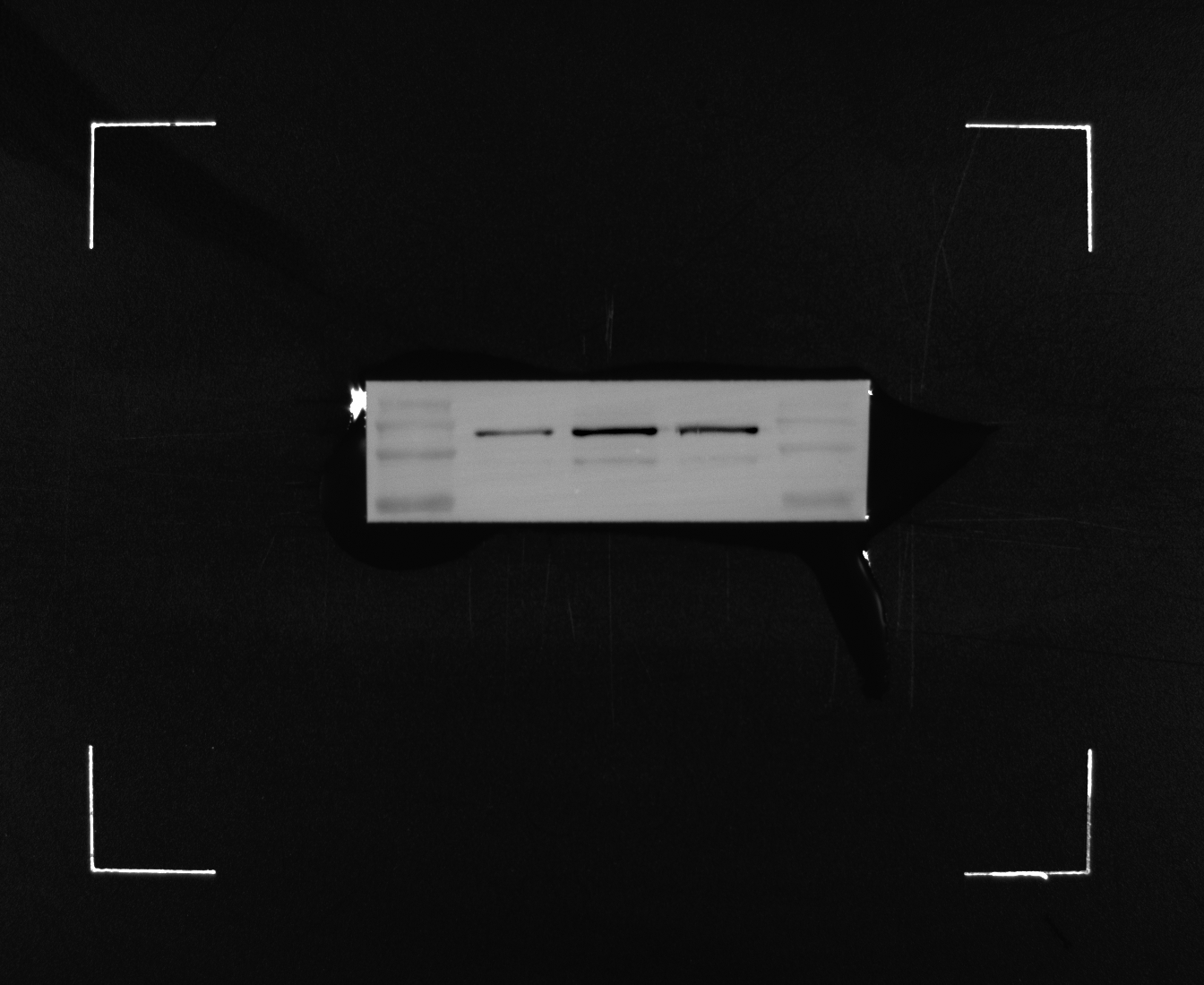

Supplement: Supplementary file 1 [file animals-14-00040-s001.zip › Westernblot/TIFF images/p-HIF-1a┴-3.tif]

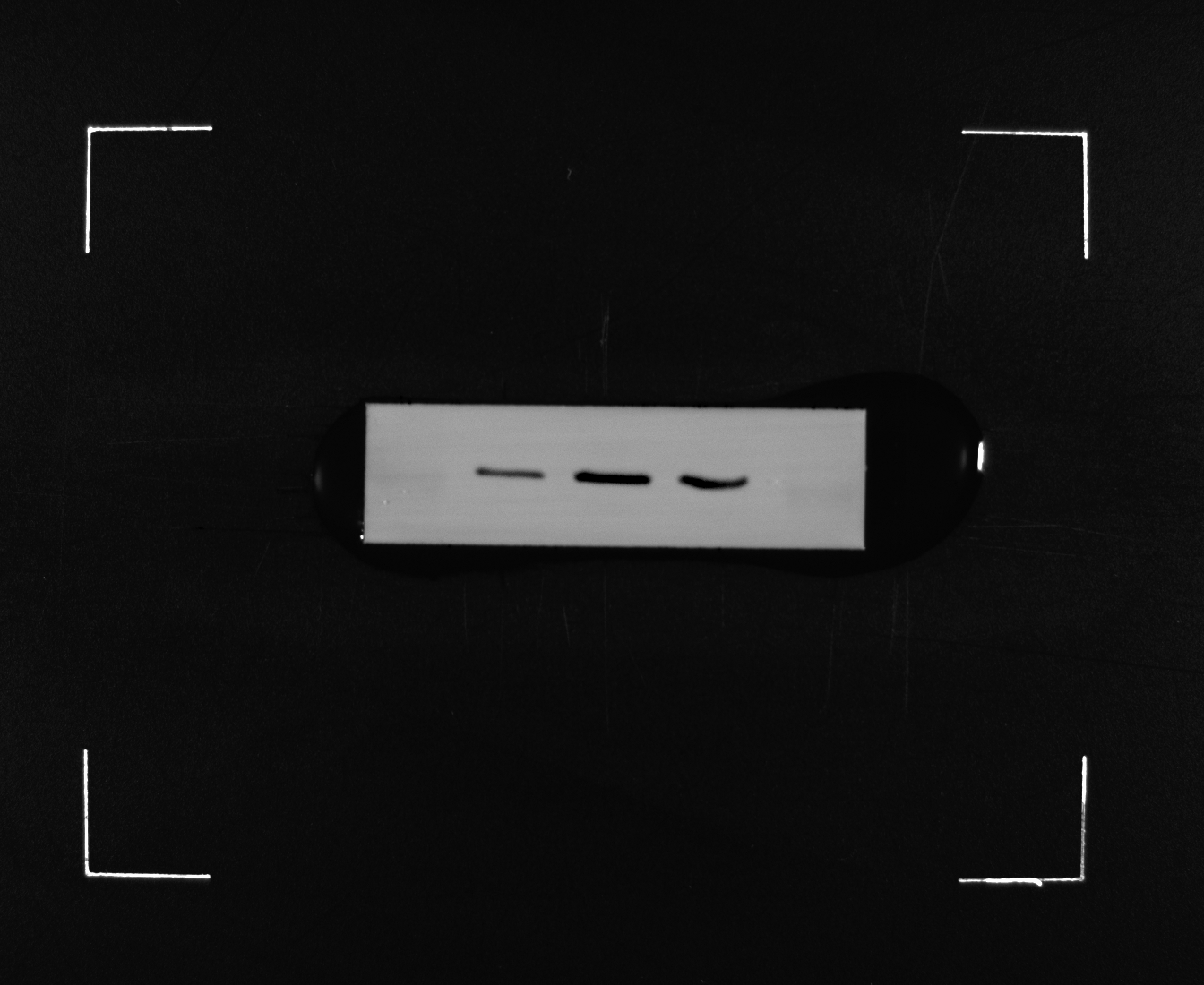

Supplement: Supplementary file 1 [file animals-14-00040-s001.zip › Westernblot/TIFF images/p-mTOR-1.tif]

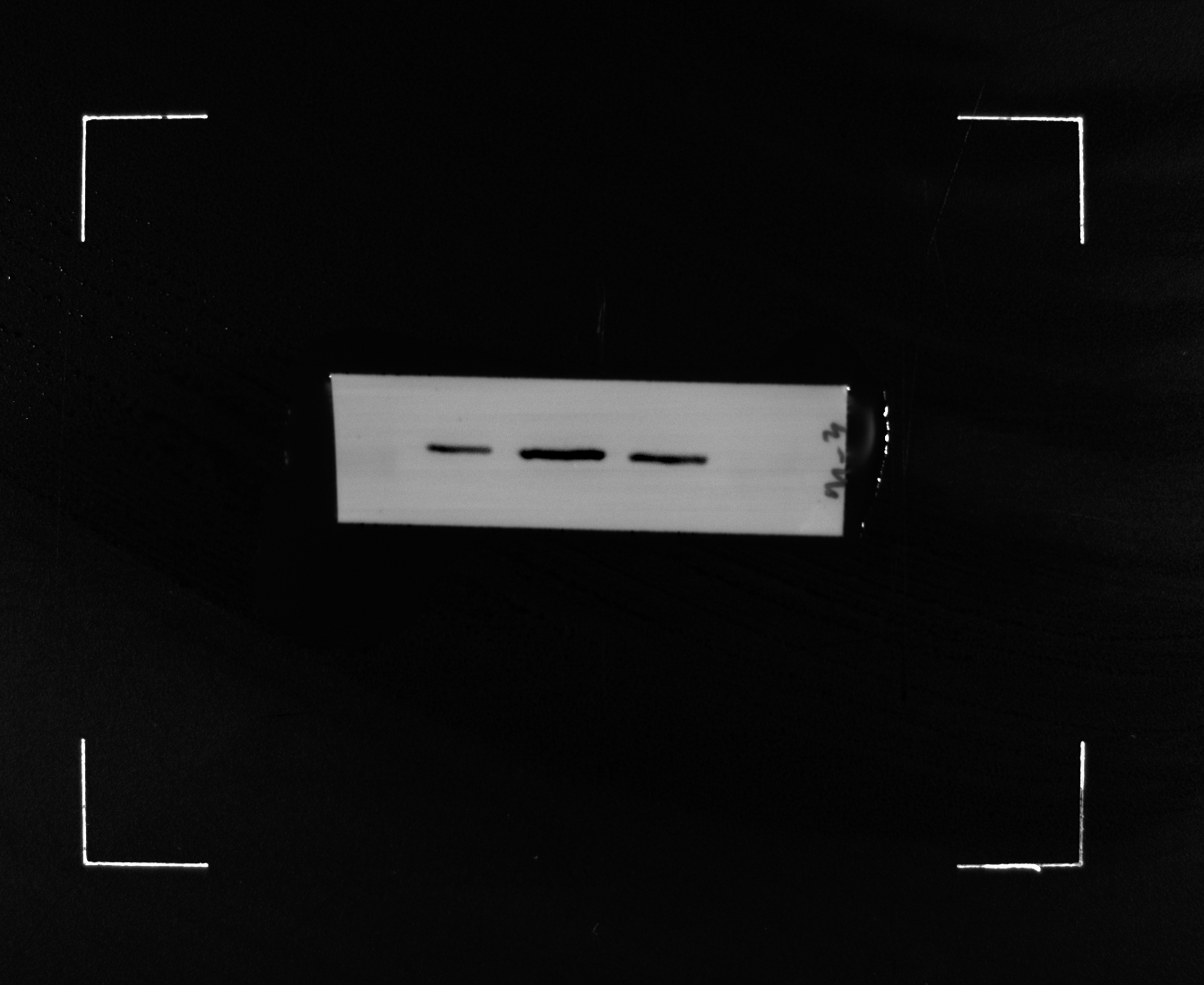

Supplement: Supplementary file 1 [file animals-14-00040-s001.zip › Westernblot/TIFF images/p-mTOR-2.tif]

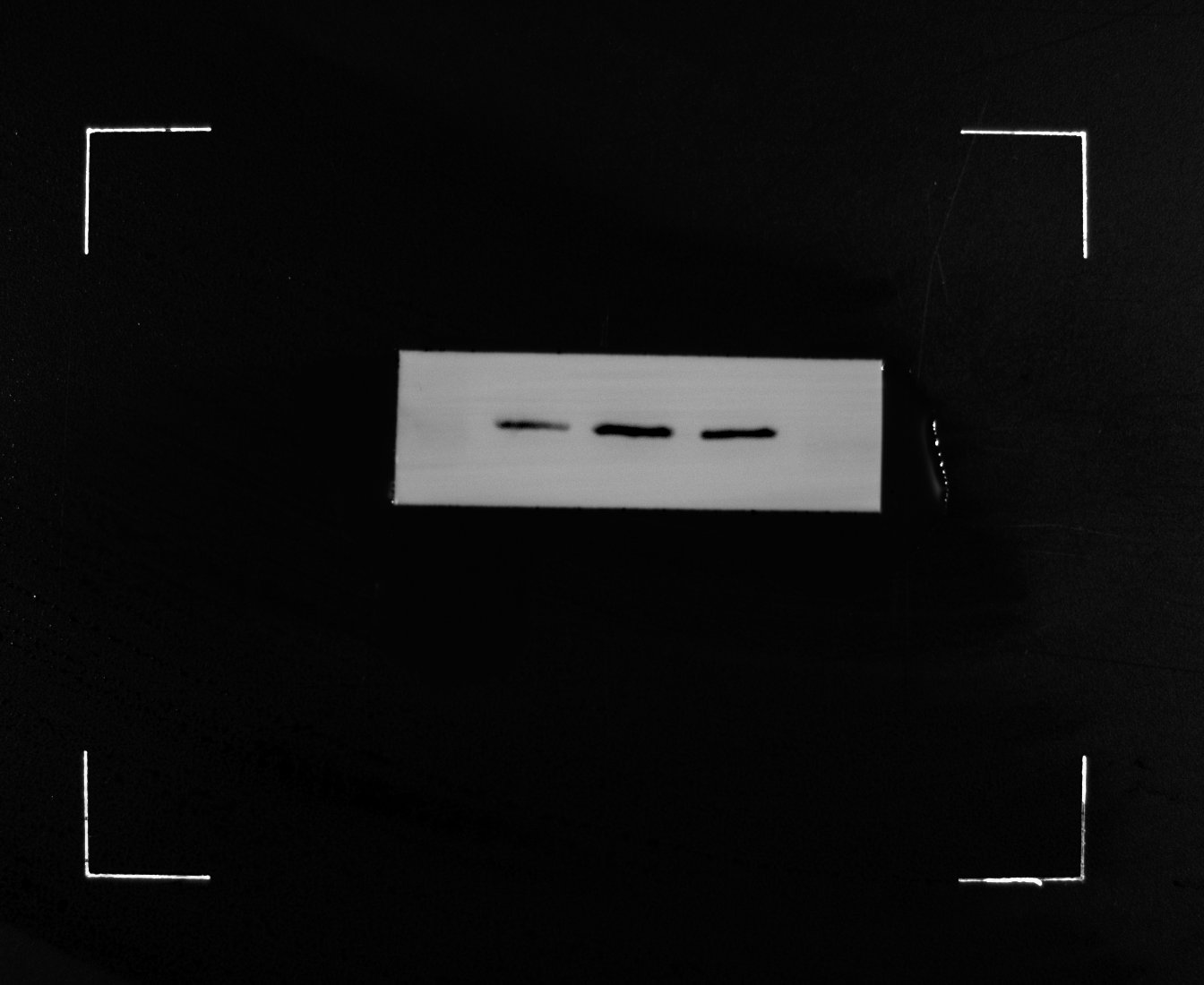

Supplement: Supplementary file 1 [file animals-14-00040-s001.zip › Westernblot/TIFF images/p-mTOR-3.tif]

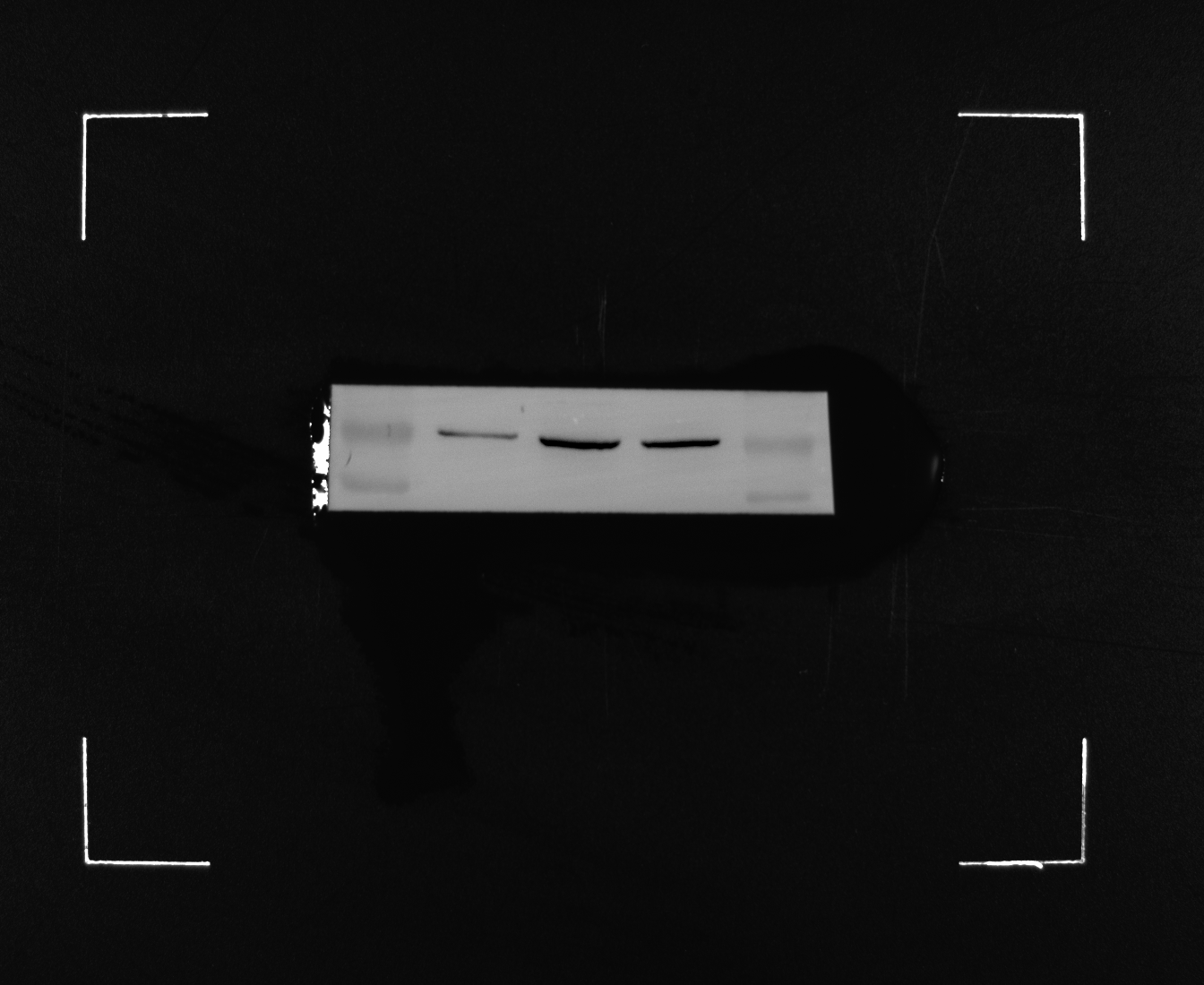

Supplement: Supplementary file 1 [file animals-14-00040-s001.zip › Westernblot/TIFF images/p-P70S6K-1.tif]

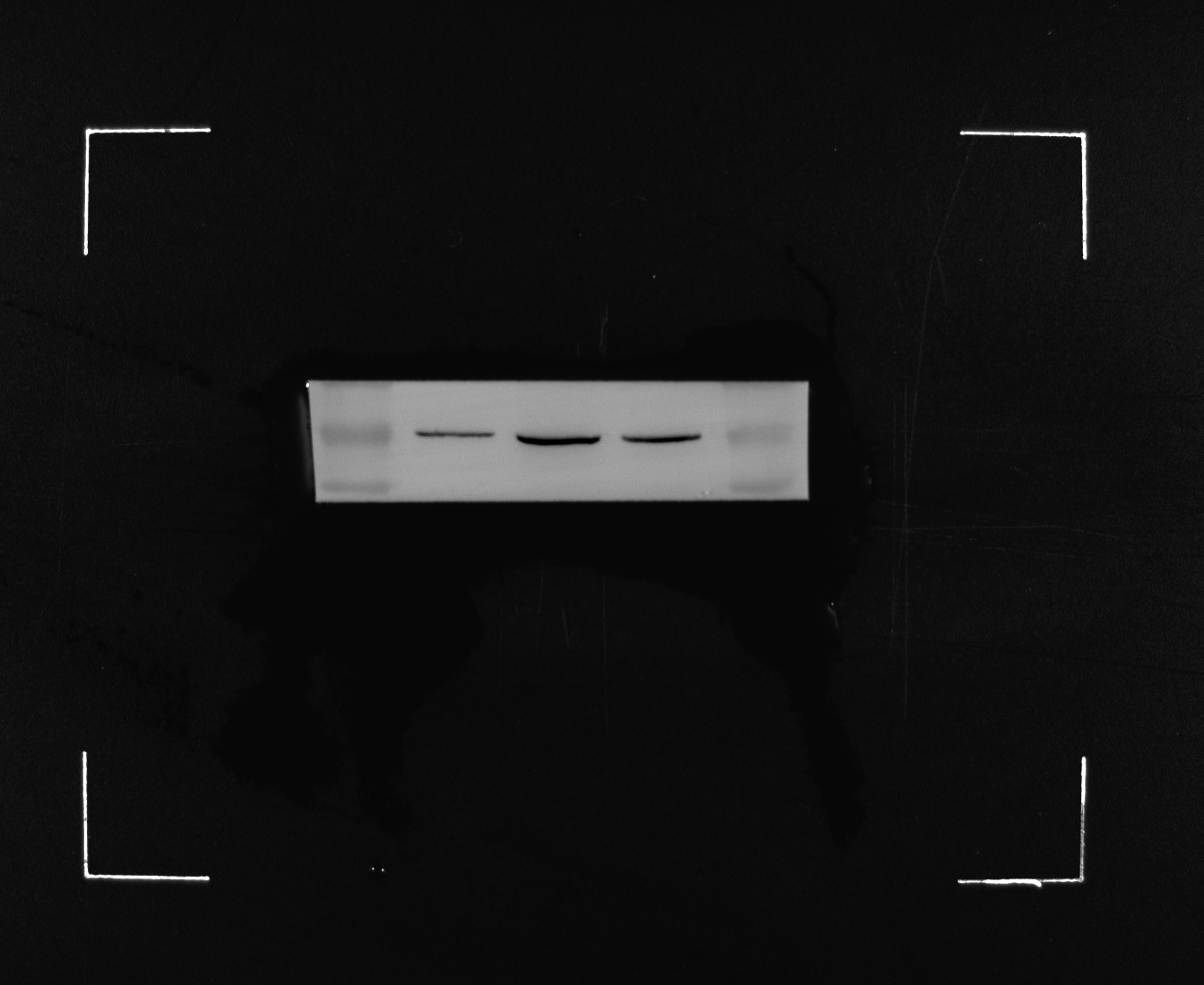

Supplement: Supplementary file 1 [file animals-14-00040-s001.zip › Westernblot/TIFF images/p-P70S6K-2.tif]

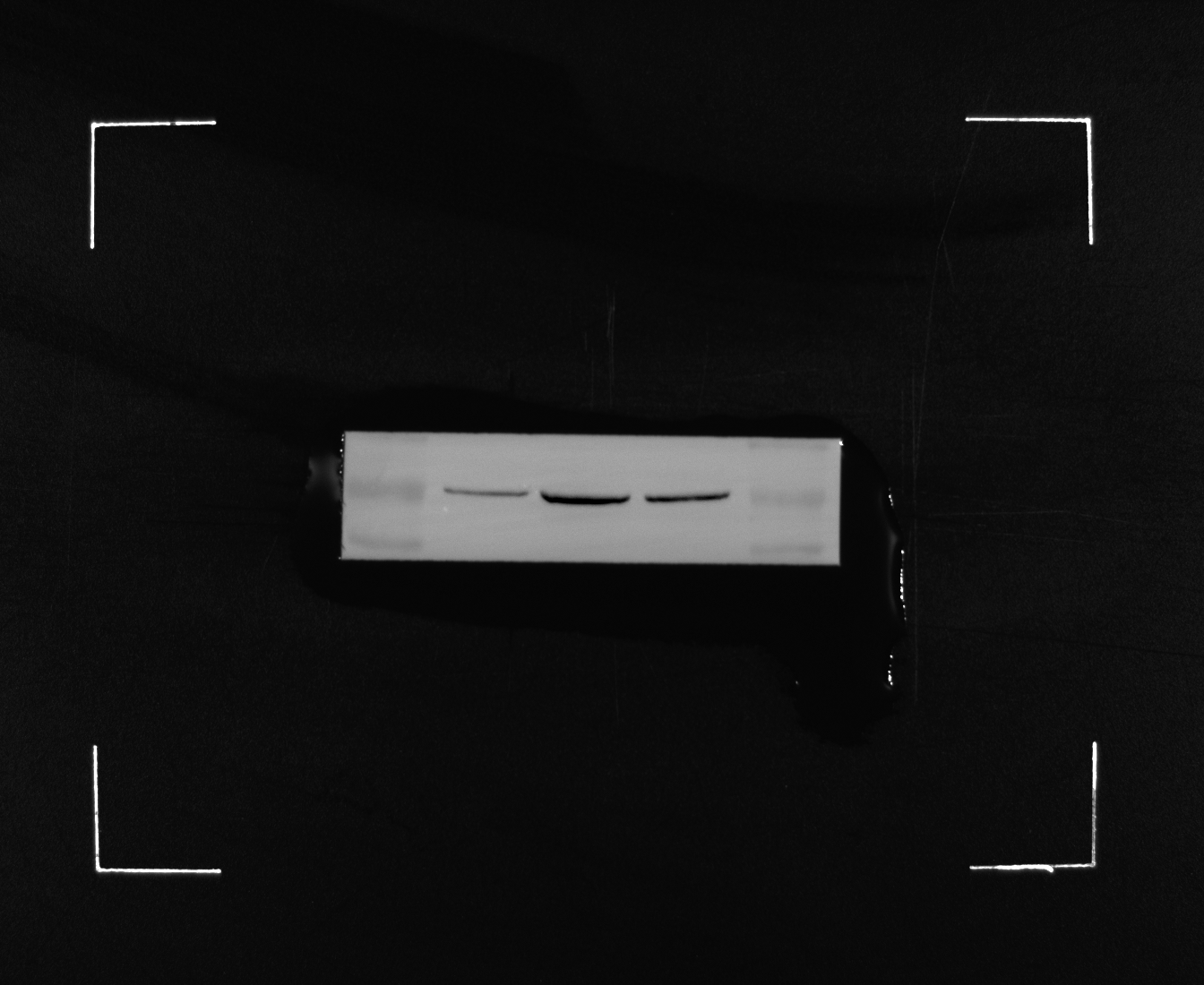

Supplement: Supplementary file 1 [file animals-14-00040-s001.zip › Westernblot/TIFF images/p-P70S6K-3.tif]

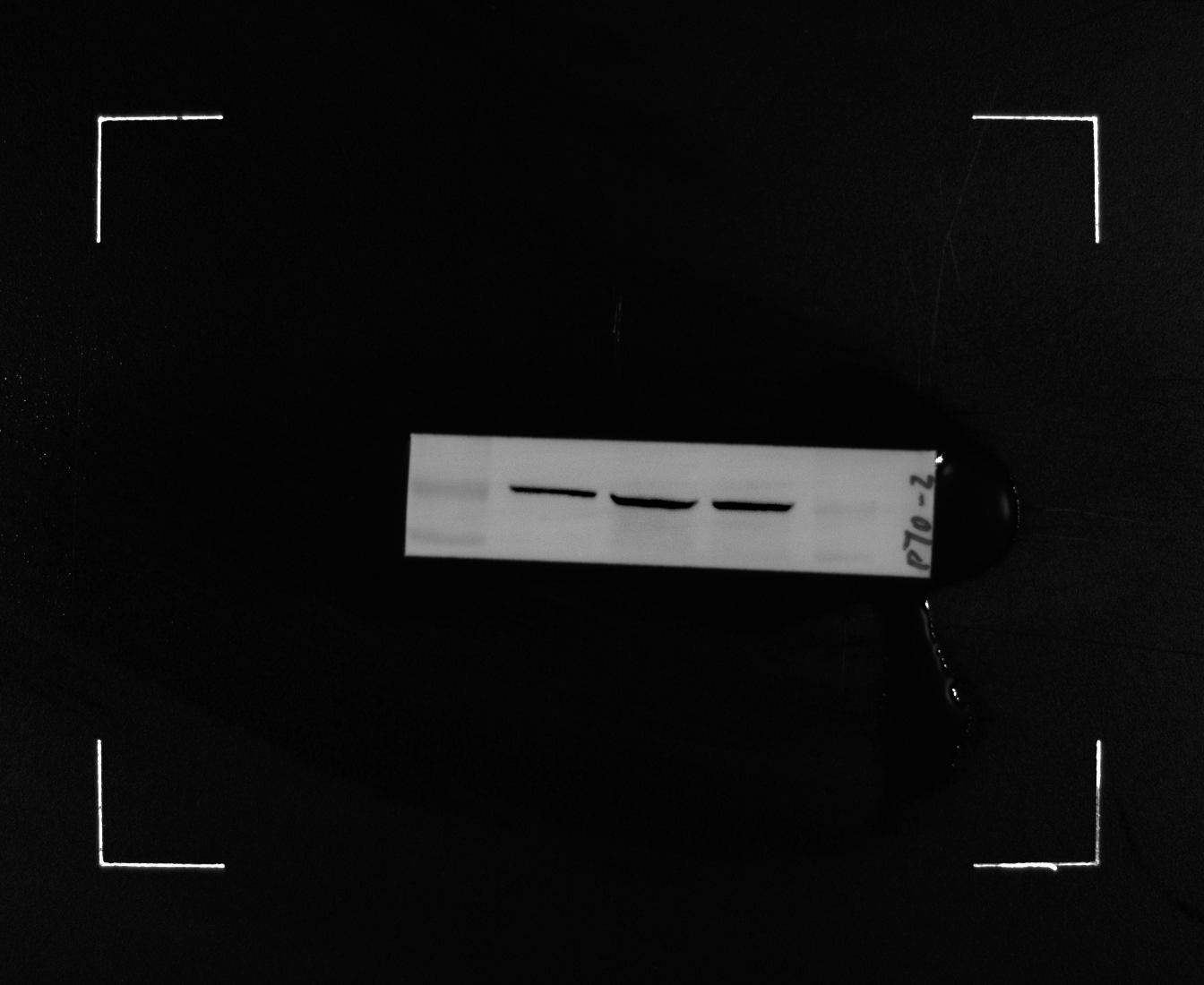

Supplement: Supplementary file 1 [file animals-14-00040-s001.zip › Westernblot/TIFF images/P70S6K-1.tif]

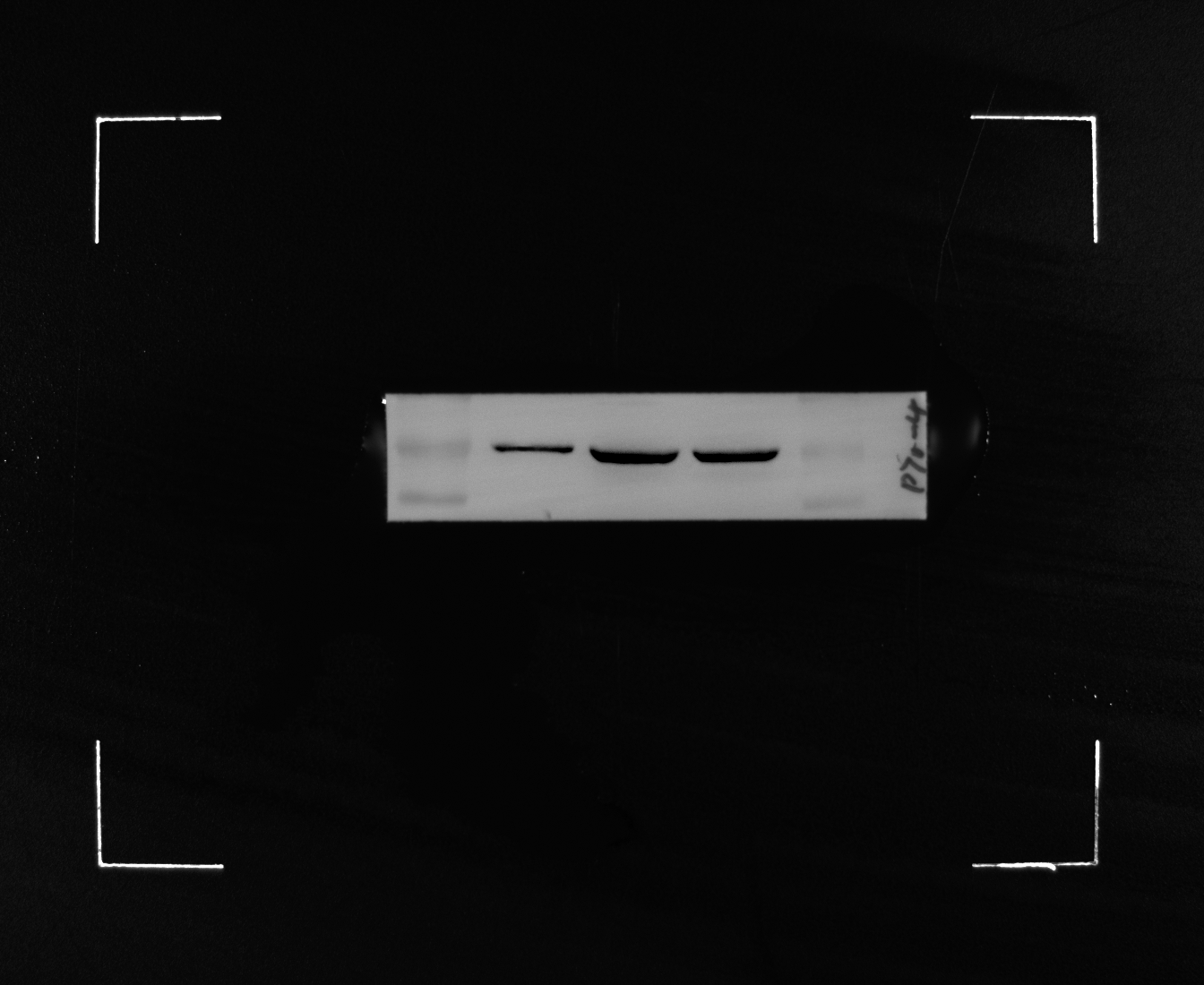

Supplement: Supplementary file 1 [file animals-14-00040-s001.zip › Westernblot/TIFF images/P70S6K-2.tif]

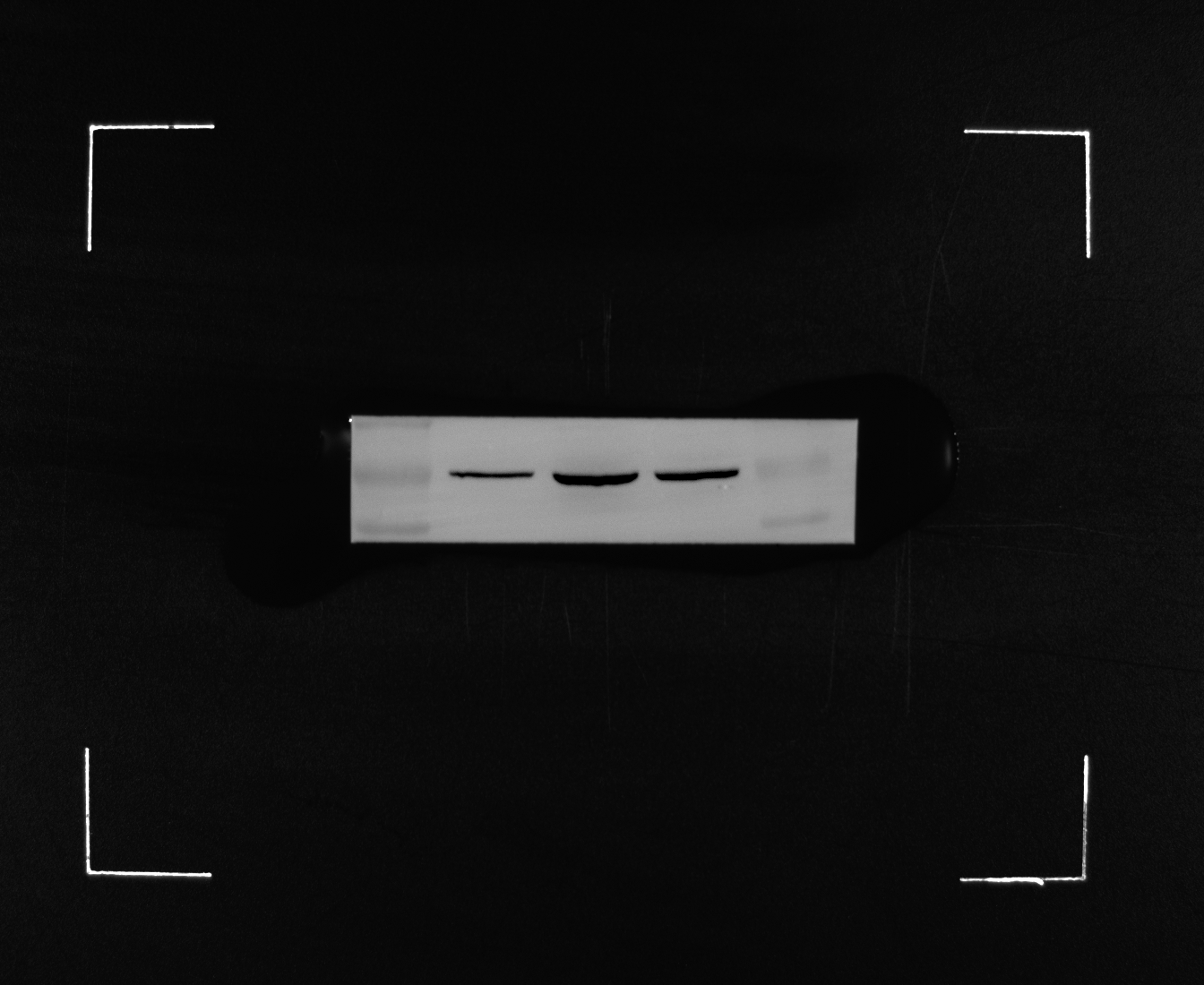

Supplement: Supplementary file 1 [file animals-14-00040-s001.zip › Westernblot/TIFF images/P70S6K-3.tif]

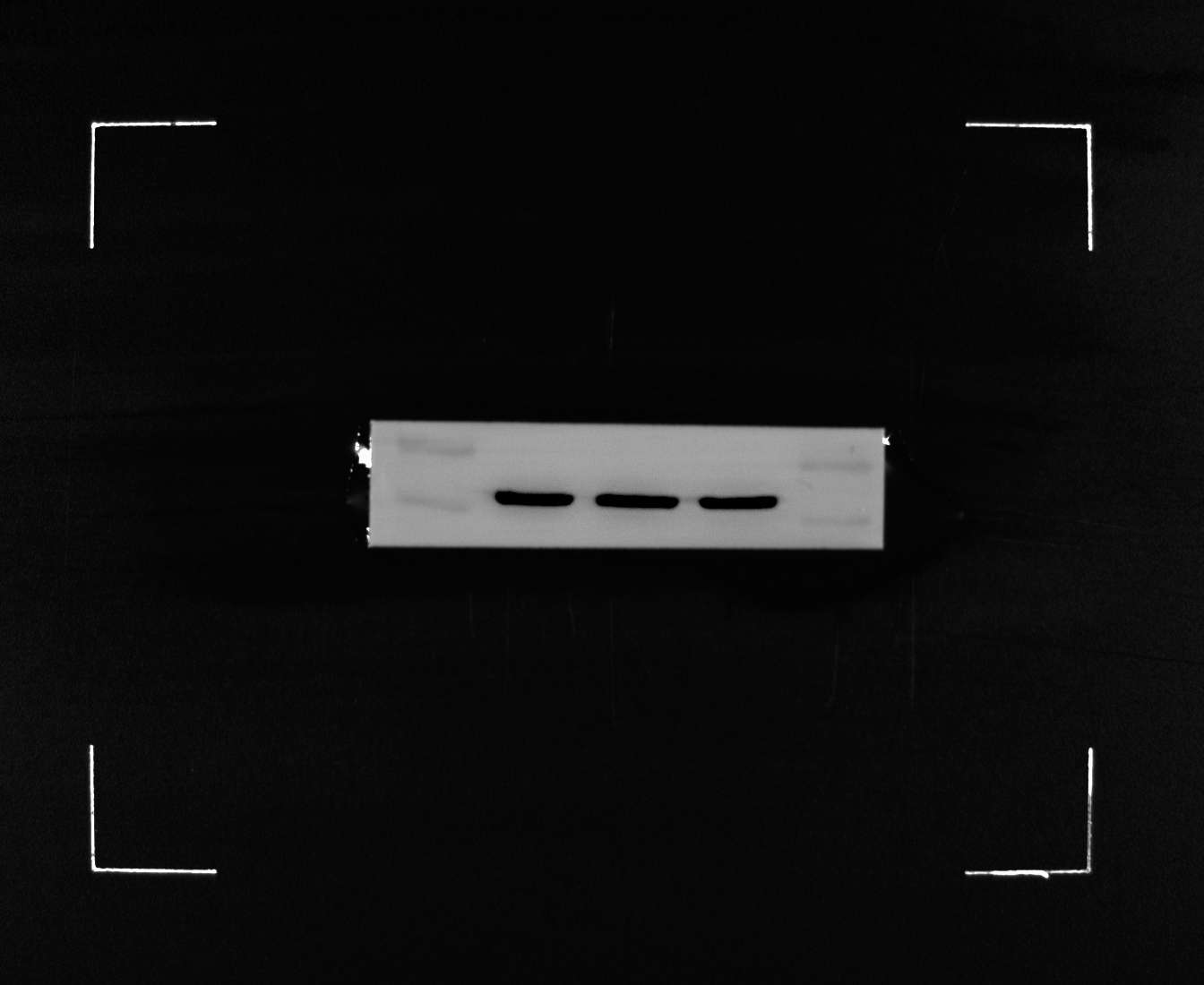

Supplement: Supplementary file 1 [file animals-14-00040-s001.zip › Westernblot/TIFF images/a┬-Actin-1.tif]

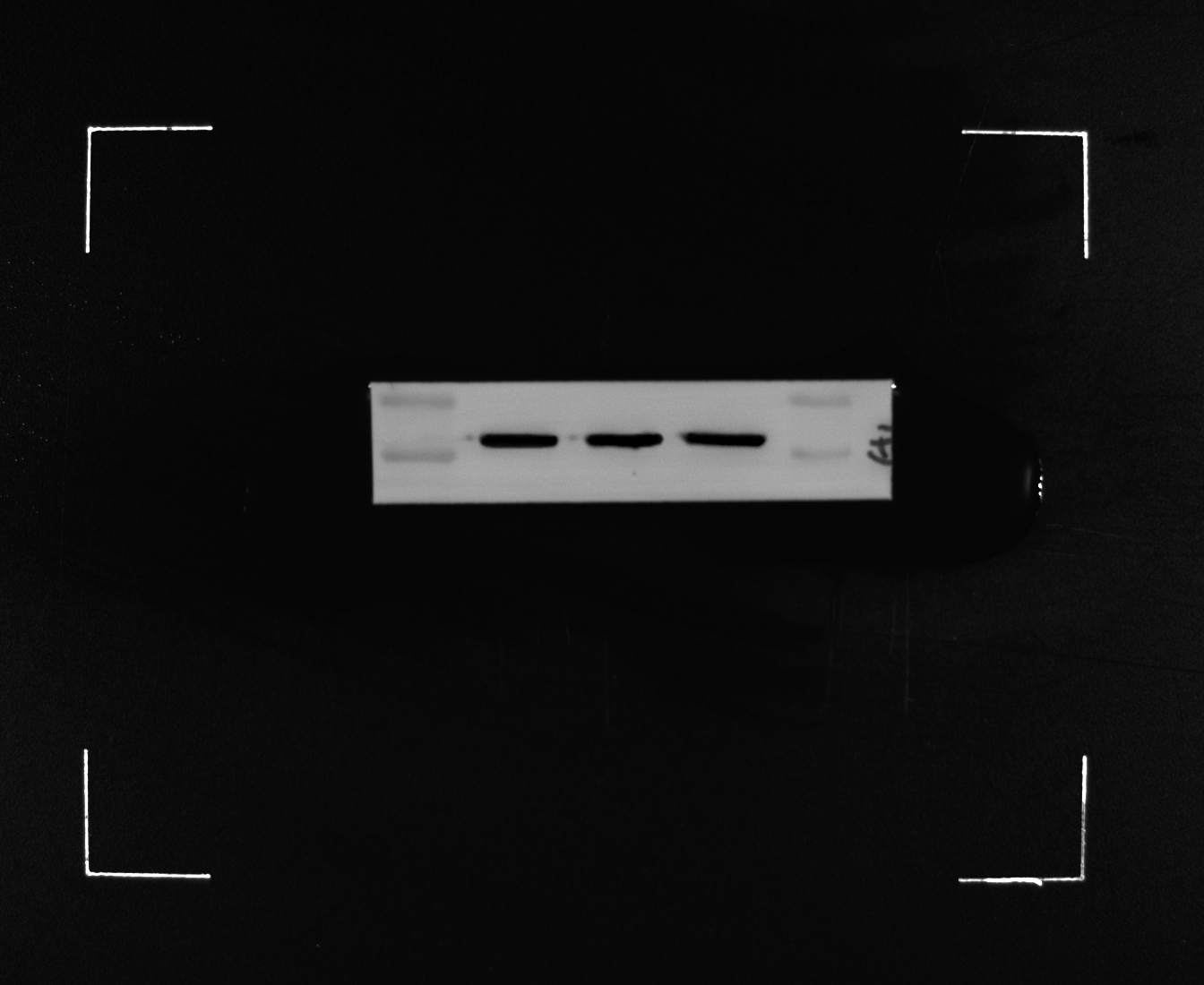

Supplement: Supplementary file 1 [file animals-14-00040-s001.zip › Westernblot/TIFF images/a┬-Actin-2.tif]

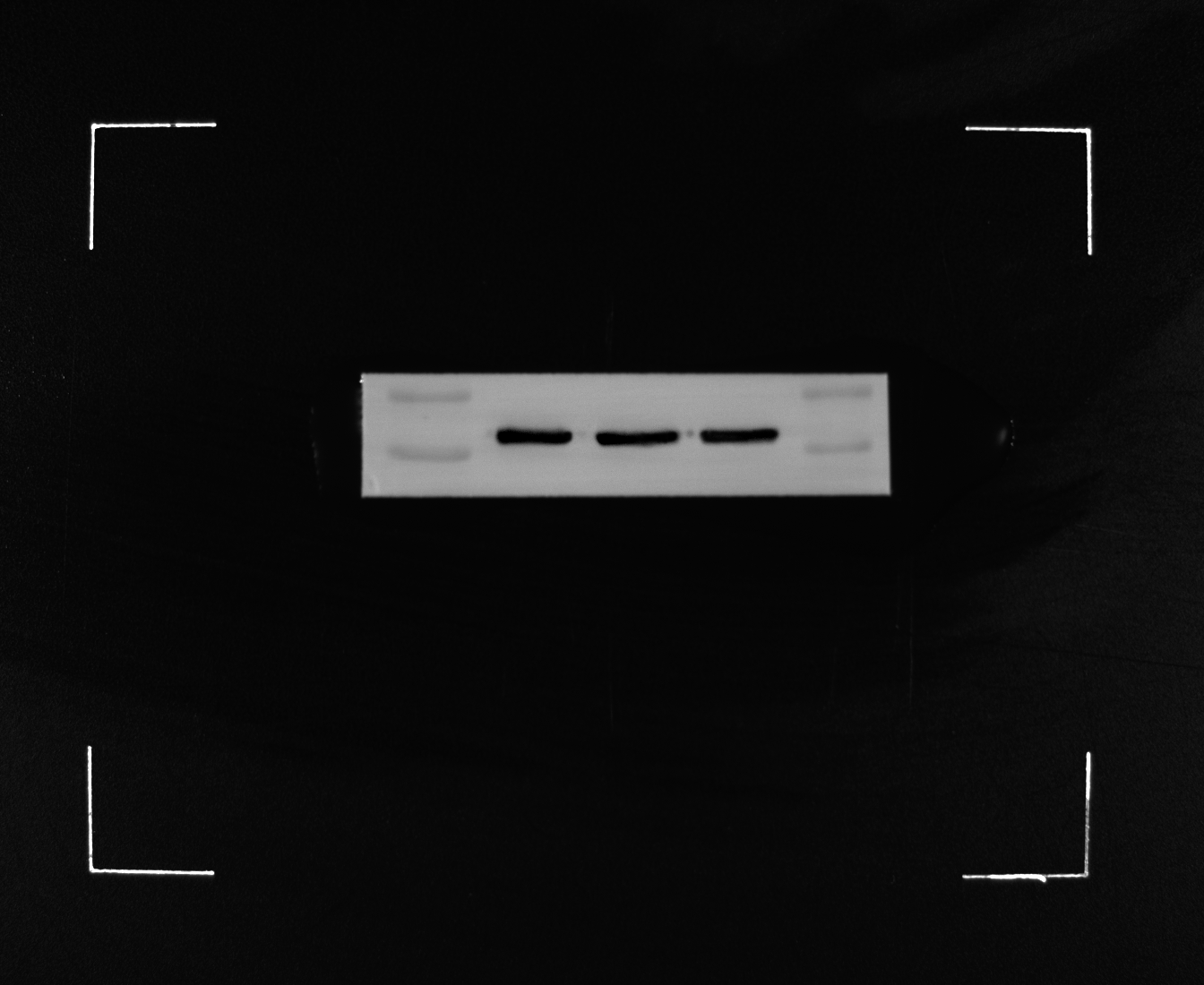

Supplement: Supplementary file 1 [file animals-14-00040-s001.zip › Westernblot/TIFF images/a┬-Actin-3.tif]

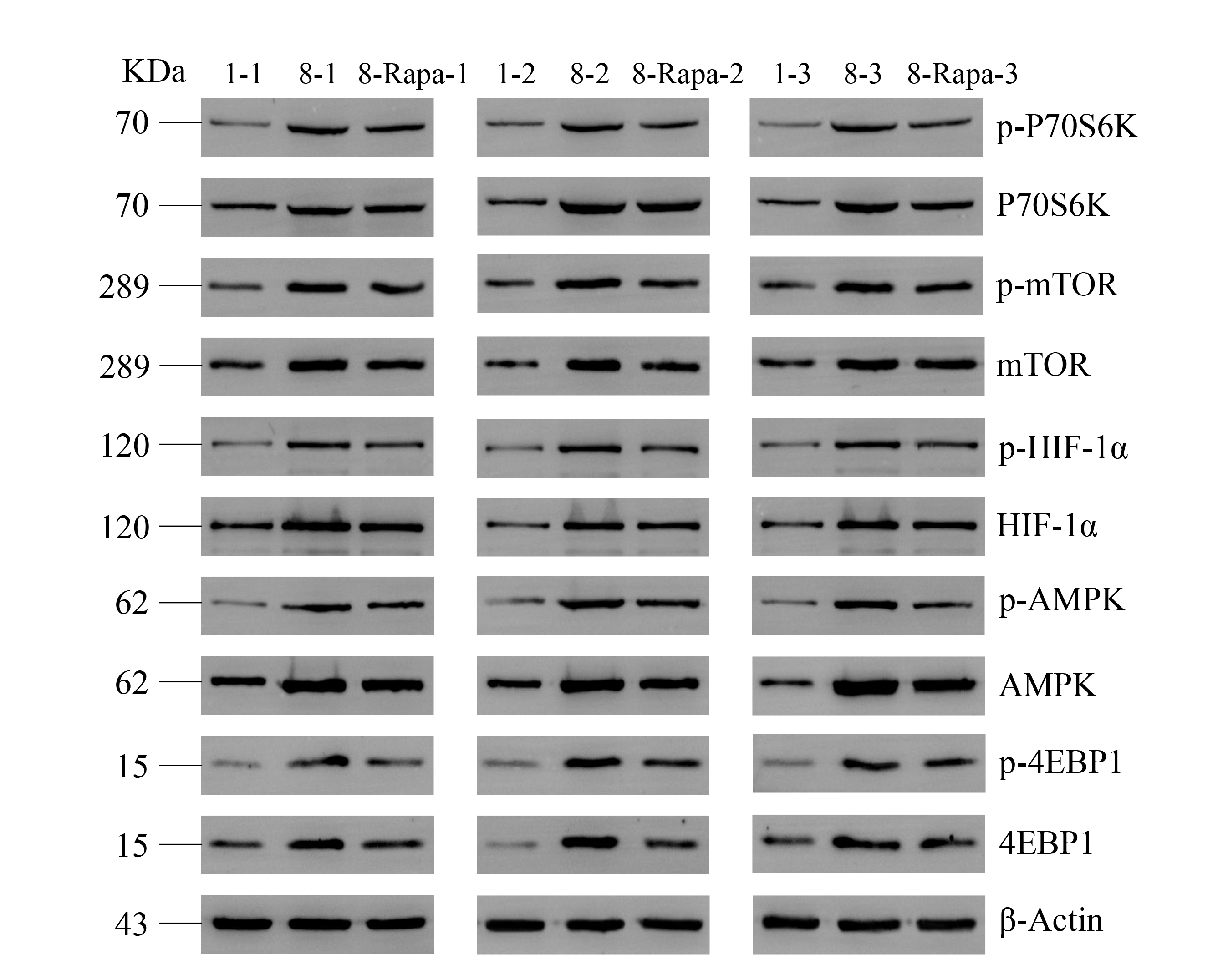

Supplement: Supplementary file 1 [file animals-14-00040-s001.zip › Westernblot/WB.tif]
